# Ein Instrument zur Erfassung biopsychosozialer Schmerzkonzepte von Erwachsenen: Entwicklung und Evaluation durch Expert*innen

**DOI:** 10.1007/s00482-024-00793-2

**Published:** 2024-02-13

**Authors:** L. Wickering, C. Lautwein, A. Fiegler, L. Allerdißen, T. Kloos, M. Schneider, T. Hechler

**Affiliations:** Arbeitseinheit Klinische Psychologie des Kindes- und Jugendalters, Universität Münster, Fliednerstr. 21, 48149 Münster, Deutschland

**Keywords:** Schmerzwissen, Psychoedukation, Fragebogenentwicklung, Multidimensionale Schmerzerfassung, Chronische Schmerzen, Pain knowledge, Psychoeducation, Questionnaire development, Multidimensional pain assessment, Chronic pain

## Abstract

**Hintergrund:**

Das biopsychosoziale Modell ist Grundlage für das Verständnis und die Behandlung chronischer Schmerzen. Ob Betroffene ein biopsychosoziales Verständnis zeigen, ist wenig erforscht. Hier wird der multidimensionale Fragebogen „Biopsychosoziale Schmerzkonzept Matrix“ (BiPS Matrix) vorgestellt. Die Konzeption der BiPS Matrix basiert sowohl auf den Bereichen biologisch, psychologisch und sozial als auch auf dem Common-Sense Model of Self-Regulation mit fünf Dimensionen: (1) Art der Störung, (2) Annahmen zur Ursache, (3) Konsequenzen, (4) zeitlicher Krankheitsverlauf, (5) Möglichkeiten der Kontrolle/Behandlung.

**Ziel:**

Ziel der vorliegenden Studie ist die Erfassung der inhaltlichen Relevanz und Verständlichkeit der Bereiche, Dimensionen und Items inklusive der Verwendung der BiPS Matrix mit Kindern durch interdisziplinäre Expert*innen-Ratings. Perspektivisch kann der Fragebogen von Behandelnden im Rahmen der Diagnostik angewendet werden.

**Methode:**

In einer Online-Studie wurden 17 Expert*innen befragt. Neben deskriptiven Statistiken wurden Kommentare mittels qualitativer Inhaltsanalyse nach Mayring ausgewertet.

**Ergebnisse:**

Alle Expert*innen schätzten die Erfassung von Schmerzkonzepten mit der BiPS Matrix sowie die Bereiche und Dimensionen als sehr relevant ein. Hinsichtlich der Items wurden vorwiegend Vorschläge bezüglich der Itemformulierungen sowie Anpassungsvorschläge für Kinder gemacht.

**Diskussion:**

Die BiPS Matrix stellt aus Expert*innensicht ein relevantes Instrument dar. Weitere Forschung zur psychometrischen Überprüfung der BiPS Matrix bei Erwachsenen und Kindern ist indiziert. Daneben kann die BiPS Matrix auch zur Erforschung von Schmerzkonzepten an medizinischen und psychotherapeutischen Berufsgruppen zur Abbildung des biopsychosozialen Schmerzverständnisses und der damit einhergehenden Behandlungsangebote eingesetzt werden.

**Zusatzmaterial online:**

Die Online-Version dieses Artikels (10.1007/s00482-024-00793-2) enthält die soziodemografischen Daten der Proband*innen der ersten Erprobung der BiPS Matrix, alle für die Expert*innenbefragung genutzten Fragebögen, den Codierleitfaden für die qualitative Datenauswertung ,ausführlichere Daten der vorliegenden Studie und die revidierte Version der BiPS Matrix.

## Abstract (en)

**Background:**

The biopsychosocial model is fundamental for the understanding and treatment of chronic pain; however, little research has focused on whether those affected show a biopsychosocial understanding. The multidimensional questionnaire Biopsychosocial Pain Concept Matrix (BiPS matrix) is presented. The conception of the BiPS matrix is based on the biological, psychological and social areas as well as on the common sense model of self-regulation with five dimensions: (1) type of disorder, (2) assumptions on the causes, (3) consequences, (4) duration of the disease and (5) possibilities for control and treatment.

**Objective:**

The present study aims to examine the content relevance and comprehension of the areas, dimensions and items, including the use of the BiPS matrix with children by interdisciplinary expert ratings. The questionnaire can be perspectively used by treating professionals for diagnostic purposes.

**Method:**

In an online study 17 experts were questioned. In addition to descriptive statistics, comments were evaluated using qualitative content analysis according to Mayring.

**Results:**

All experts rated the assessment of pain concepts with the BiPS matrix as well as the areas and dimensions as very relevant. With respect to the items, suggestions were made mainly regarding the wording of the items and adjustments for children.

**Discussion:**

From an expert point of view the BiPS matrix represents a relevant instrument. Further research on the psychometric properties of the BiPS matrix in adults and children is indicated. In addition, the BiPS matrix can also be used to investigate pain concepts of medical and psychotherapeutic professional groups to demonstrate the biopsychosocial understanding of pain and the associated treatment options.

## Hintergrund und Fragestellung

Chronische (primäre) Schmerzen stellen ein zentrales Problem der Gesundheitsversorgung dar [12]. Subjektive Schmerzkonzepte spielen bei der Behandlung eine wichtige Rolle [19]. Messinstrumente zur Erfassung dieser Konzepte sind rar, könnten jedoch zur Identifikation von Wissenslücken und Misskonzepten von Betroffenen beitragen. Die hier vorgestellte Biopsychosoziale Schmerzkonzept Matrix (BiPS Matrix) stellt ein innovatives Tool dar, mit dem zukünftig die Erfassung von biopsychosozialen Schmerzkonzepten zur Umsetzung einer individualisierten Psychoedukation möglich wird.

Chronische (primäre) Schmerzen gehen mit einer anhaltenden psychosozialen Beeinträchtigung, einer eingeschränkten Lebensqualität sowie erhöhten Gesundheitskosten einher [8, 31]. Insbesondere im Forschungskontext ist das biopsychosoziale Erklärungsmodell [7] für das Verständnis und die Behandlung von chronischem Schmerz zentral. Nach diesem Ansatz sind komplexe, dynamische Wechselwirkungen zwischen biologischen, psychologischen und sozialen Faktoren für die Entstehung und Aufrechterhaltung chronischer Schmerzen verantwortlich [7]. Trotz des kontinuierlichen Erkenntnisgewinns bezüglich des biopsychosozialen Modells chronischer (primärer) Schmerzen konnte gezeigt werden, dass über 60 % der Betroffenen mit chronischem Schmerz keine multimodale Schmerztherapie erhalten [5]. Die Versorgung von Kindern und Erwachsenen erscheint daher nach wie vor unzureichend [10, 11].

Eine Schlüsselfrage ist daher, welche Faktoren zu der unzureichenden Versorgung und zum maladaptiven Umgang mit chronischen Schmerzen beitragen. Ein ausschlaggebender Faktor in Bezug auf chronische Schmerzen sind die Schmerzkonzepte der Betroffenen sowie der versorgenden Berufsgruppen [1].

Pate et al. [25] definieren, basierend auf der Arbeit von Moseley und Butler [20], ein Schmerzkonzept als das Verständnis einer Person davon, was Schmerz ist, welche Funktion Schmerz hat und welche biologischen Prozesse zu dessen Entstehung und Aufrechterhaltung beitragen. Schmerzkonzepte sind von Schmerzüberzeugungen, sog. „pain beliefs“, abzugrenzen. Kröner-Herwig [14, S. 264] definiert „pain beliefs“ als „ungünstige Überzeugungen von Schmerzpatient*innen“ (z. B. „am besten hinlegen und schonen“). Es handelt sich hierbei um dogmatische Einstellungen und Überzeugungen über die Schmerzätiologie und Schmerzfolgen, die das Befinden und Verhalten der Person dysfunktional beeinflussen. Somit werden „pain beliefs“ als persönliches Verständnis der eigenen Schmerzerfahrung definiert (vgl. [33, S. 351]). Chi [4] definiert „beliefs“ als singuläre Ideen. Konzepte hingegen werden definiert als übergreifendes mentales Wissenskonstrukt bestehend aus korrekten und inkorrekten Teilaspekten [21]. Daraus können Misskonzepte resultieren, die mit korrekten wissenschaftlichen Konzepten konkurrieren, Erklärungen liefern und Zusammenhänge aufdecken. Das Erlernen korrekter Konzepte wird durch vorhandene Misskonzepte erschwert [32]. Es konnte gezeigt werden, dass insbesondere chronische Schmerzpatient*innen ein biologisches Konzept ihrer Schmerzen aufweisen [13], z. B. „Mit der heutigen Technik sollten die Ärzte doch in der Lage sein, das Problem zu beheben“ (vgl. [21, S. 114–115]). In der vorliegenden Arbeit liegt der Fokus auf der Erfassung der Schmerzkonzepte, wie z. B. der Annahme, dass die Schmerzstärke mit dem Ausmaß der Gewebeschädigung assoziiert ist [19].

Konzepte von Krankheit lassen sich nach Hagger und Orbell [9] in verschiedene Inhaltsbereiche einteilen. Das sogenannte Common-Sense Modell of Self Regulation (CSM), eines der bekanntesten Modelle in diesem Bereich, umfasst fünf Inhaltsbereiche: (1) Art der Störung, (2) Annahmen zur Ursache, (3) Konsequenzen der Krankheit für die Betroffenen, (4) zeitlicher Krankheitsverlauf und (5) Möglichkeiten der Kontrolle/Behandlung. Die erste Dimension „Art der Störung“ umfasst das Krankheitslabel und die mit der jeweiligen Krankheit assoziierten Symptome. „Annahmen zur Ursache“ (zweite Dimension) der Schmerzen könnten zum Beispiel biologischer Art (z. B. Bakterien, Viren) oder emotionaler Art (z. B. Stress, Depression) sein. Bei der dritten Dimension „Konsequenzen der Krankheit für die Betroffenen“ handelt es sich um die Annahmen über die Auswirkungen der Krankheit auf die Lebensqualität und das allgemeine Funktionsniveau. Die vierte Dimension „zeitlicher Krankheitsverlauf“ bezieht sich auf Vorstellungen zur relativen Chronizität und Konstanz einer Krankheit. Die fünfte Dimension „Möglichkeiten der Kontrolle und Behandlung“ umfasst die Einschätzung der eigenen Handlungsfähigkeit, die Kontrollmöglichkeiten (etwa die regelmäßige Einnahme von Medikamenten) und die Wirksamkeit von Behandlungen. Moss-Morris und Kolleg*innen [23] untersuchten die Inhaltsdimensionen von Schmerzpatient*innen mit akuten und chronischen Schmerzen mithilfe des Illness Perception Questionnaire (IPQ-R). Sie konnten zeigen, dass Personen mit chronischem Schmerzleiden zum einen ein inadäquateres, also weniger kohärentes Schmerzverständnis hatten als Personen mit akutem Schmerzleiden und zum anderen die Annahme vertraten, weniger Kontrolle über die eigenen Schmerzen zu haben, und mit schlimmeren Konsequenzen rechneten.

Auf Basis der Erkenntnisse zur biopsychosozialen Ätiologie und Aufrechterhaltung von chronischem Schmerz und der Differenzierung in die fünf Inhaltsbereiche nach dem CSM erscheint es sinnvoll, beide Ansätze zu kombinieren und bei der Erfassung von Schmerzkonzepten zu integrieren. Eine offene Frage ist, inwieweit die Schmerzkonzepte Betroffener mit dem biopsychosozialen Modell übereinstimmen oder von diesem wissenschaftlichen, allgemein akzeptierten Standard abweichen. In bisherigen Untersuchungen mehren sich Hinweise, dass Erwachsene und insbesondere chronische Schmerzpatient*innen häufig ein vorwiegend biomedizinisches Modell ihrer Schmerzen haben [13]. Aktuell existieren jedoch nur zwei internationale Fragebögen, die Schmerzkonzepte Erwachsener erfassen.

*Neurophysiology of Pain Questionnaire* (NPQ) [22]. Der NPQ untersucht das neurophysiologische Schmerzwissen bei Erwachsenen [30]. Die deutsche Version des NPQ (NPQ‑d; [30]) beinhaltet 12 Items, die dichotom bewertet werden. In der Praxis wird der NPQ zur Erfassung von Schmerzkonzepten bei Schmerzpatient*innen genutzt, um Wissenslücken und intuitive Konzepte zu identifizieren und diese durch individuelle schmerzedukative Maßnahmen zu verändern [3]. Des Weiteren wird er als Instrument der Veränderungsmessung eingesetzt [18]. Allerdings erfasst der NPQ – anders als die BiPS Matrix – lediglich das neurophysiologische Schmerzwissen Erwachsener und weist keine mehrdimensionale Struktur auf.

*Concept of Pain Inventory* (COPI; [26]). Pate et al. [26] entwickelten das auf dem biopsychosozialen Ansatz basierende *Concept of Pain Inventory* (COPI), um Schmerzkonzepte von Kindern zwischen acht und zwölf Jahren zu erfassen. Dieser Fragebogen wurde 2021 für Erwachsene adaptiert [27]. Das COPI für Erwachsene erfasst Schmerzkonzepte mithilfe von 13 Items auf einer 5‑Punkte-Likert-Skala von 0 „stimme überhaupt nicht zu“ bis 4 „stimme stark zu“ (z. B. Item 1: „Feeling sad can make you feel more pain.“). Das COPI erfasst zwar psychologische und biologische Aspekte chronischer Schmerzen, vernachlässigt jedoch soziale Aspekte, welche einen wichtigen Bestandteil im Verständnis von Schmerzkonzepten bilden: Die Schmerzen führen nicht nur dazu, dass sich Betroffene vermehrt zurückziehen, sondern auch dazu, dass sich Interaktionen mit Bezugspersonen auf die Schmerzen fokussieren und in der Folge die Beziehung belastet wird [6, 24].

Die kombinierte Erfassung der biopsychosozialen Schmerzkonzepte und der nach CSM postulierten Inhaltsdimensionen wurde in keinem der beiden Tools angewandt. Demnach existiert aktuell kein deutschsprachiges Instrument, das biopsychosoziale Aspekte des Schmerzkonzepts basierend auf den postulierten Inhaltsbereichen des CSM vollständig, mehrdimensional und standardisiert erfasst. Mithilfe eines solchen Instruments wäre es möglich, dass Behandelnde die Schmerzkonzepte Erwachsener auf biologischer, psychologischer und sozialer Ebene über die fünf Inhaltsbereiche des CSM [9] hinweg erfassen [13]. Eine detaillierte Erfassung der Schmerzkonzepte kann dazu beitragen, dass auch Behandelnde durch den Einsatz der BiPS Matrix in der Therapie für ein biopsychosoziales Schmerzverständnis sensibilisiert werden. So können sie ihre Behandlung individualisiert nach dem Schmerzkonzept ihrer Patient*innen ausrichten. Parallel profitieren die Patient*innen von einer individuell angepassten psychoedukativen Intervention, die an ihre Schmerzkonzepte bzw. Misskonzepte anknüpft.

Ziel dieser Arbeit ist es daher, erstmals ein Instrument, welches diesen Anforderungen genügt (biopsychosoziale Aspekte, CSM), vorzustellen und anhand von Expert*innen-Ratings zu evaluieren, da diese ein Standardverfahren für die Überprüfung der Eignung der Fragebogenstruktur und Iteminhalte darstellen. Expert*innen besitzen hohes Fachwissen in spezifischen Wissensbereichen, hier operationalisiert durch eine mindestens zweijährige Berufserfahrung im Bereich chronischer Schmerzen bzw. durch eine Beteiligung an aktueller Forschung (mindestens zwei Forschungsarbeiten in den letzten fünf Jahren). Durch dieses verfügbare Fachwissen können die Expert*innen die Passung der konzipierten Items zu den Bereichen bewerten. Anhand der Bewertungen können neue Tools für die praktische Anwendung adaptiert werden [2, 25].

Die BiPS Matrix wurde auf Grundlage des biopsychosozialen Modells entwickelt und setzt sich aus insgesamt 63 Items zusammen^1^. In Form einer Schmerzmatrix werden spaltenweise biologische, psychologische und soziale Schmerzkonzepte erfasst sowie zeilenweise die fünf Inhaltsbereiche des CSM abgebildet (vgl. Tab. 2). Folgende Parameter wurden von den Expert*innen geratet: (1) Relevanz der Erfassung von Schmerzkonzepten im Allgemeinen, im klinischen Kontext und im Forschungskontext, (2) Relevanz der Erfassung der drei Dimensionen (biologisch, psychologisch, sozial) und der fünf Inhaltsbereiche von Schmerzkonzepten, (3) Verständlichkeit der Items. Außerdem konnten die Expert*innen Änderungsvorschläge im Hinblick auf die allgemeine sprachliche Anpassung, perspektivisch auch für Kinder und Jugendliche, verfassen.

## Studiendesign und Untersuchungsmethoden

Bei der Expert*innenbefragung zur BiPS Matrix handelte es sich um eine Online-Fragebogenstudie mit querschnittlichem Design. Die Erhebung der Daten erfolgte über das Programm *Unipark *der Firma *QuestBack*. Die Expert*innenumfrage wurde im Einklang mit nationalem Recht sowie gemäß der Deklaration von Helsinki von 1975 (in der aktuellen, überarbeiteten Fassung) durchgeführt. Von allen beteiligten Expert*innen liegt eine schriftliche Einverständniserklärung vor.

### Stichprobe

Die Definition einer Person als Expert*in beinhaltete, analog zu existierenden Expert*innenbefragungen (z. B. [25]), dass diese seit mindestens zwei Jahren im Bereich chronischer Schmerzen arbeitete oder in den letzten fünf Jahren mindestens zwei Forschungsarbeiten zu dem Thema veröffentlicht hatte. Zudem wurde bei der Auswahl der Expert*innen auf Interdisziplinarität geachtet, es wurden Personen aus den Bereichen Psychologie, Medizin, Physiotherapie, Krankenpflege und Forschung kontaktiert. Die Expert*innen wurden anhand existierender Netzwerke und Fachgesellschaften, wie z. B. der Deutschen Gesellschaft für Psychologische Schmerztherapie und -forschung (DGPSF), des „principal investigators“ (TH) ausgewählt. Boateng et al. [2] berichten, dass typischerweise fünf bis sieben Experten befragt werden, wobei eine Erhöhung des Stichprobenumfangs robustere Ergebnisse hervorbringt. Von 34 angeschriebenen Expert*innen folgten 21 dem Link zur Umfrage (61,8 %), wobei zwei Personen nicht der Definition des Expertenstatus entsprachen und aus der Umfrage ausgeschlossen werden mussten und zwei weitere die Umfrage abbrachen (Rücklaufquote: 50 %). 17 Expert*innen wurden inkludiert (*M* = 53,65 Jahre, *SD* = 15,23, Altersrange 31–66 Jahre; 59 % männlich; vgl. Tab. 1).

**Tab. 1:** Soziodemografische Daten der Expert*innen (*N* = 17)

Variable	*N* = 17	%
*Höchster formeller Bildungsabschluss*		
Berufsausbildung	2	11,76
Fachhochschul‑/Universitätsabschluss	3	17,65
Promotion	8	47,06
Habilitation	4	23,53
Expert*in für den		
Klinischen Kontext	9	52,94
Forschungskontext	3	17,65
Klinischen Kontext und Forschungskontext	5	29,41
*Fachdisziplin*		
Psychologie	11	64,71
Physiotherapie	1	5,88
Medizin	4	23,53
Krankenpflege	1	5,88
*Zusatzqualifikationen*		
Keine	7	41,18
Spezielle Schmerztherapie	1	5,88
Spezielle Schmerzpsychotherapie	6	35,29
Spezielle Schmerzpsychotherapie für Kinder und Jugendliche	1	5,88
Hypnotherapie	1	5,88
Fellowship	1	5,88

### Prozedere

Die Expert*innen wurden im Zeitraum vom 03.10.2019 bis zum 25.10.2019 per E‑Mail kontaktiert und mithilfe eines Links zur Online-Umfrage weitergeleitet. Alle Expert*innen, die nicht ausdrücklich ihre Teilnahme an der Befragung bestätigten, erhielten eine Woche später sowie nach zwei weiteren Wochen eine Erinnerungs-E-Mail. Zu Beginn der Online-Umfrage füllten die Expert*innen einen soziodemografischen Fragebogen aus (vgl. Anhang A im Online-Zusatzmaterial). Im Anschluss folgte die Bearbeitung der BiPS Matrix (vgl. Anhang B im Online-Zusatzmaterial).

### Messinstrumente

#### Soziodemografischer Fragebogen

Mit einem eigens entwickelten Fragebogen wurden zum einen die soziodemographischen Daten erfasst, zum anderen wurde auf die Erfüllung der zuvor festgelegten Expert*innenkriterien und die Vorerfahrungen der Expert*innen in der Behandlung von chronischen Schmerzen eingegangen.

#### Die Biopsychosoziale Schmerzkonzept Matrix (BiPS Matrix)

Die BiPS Matrix erfasst spaltenweise biologische, psychologische und soziale Schmerzkonzepte sowie zeilenweise die fünf Inhaltsbereiche von Schmerzkonzepten (vgl. Tab. 2). Grundlage für die Items war die Definition von Schmerzkonzepten, wobei die Subskalen von Expert*innen (TH, MS) stichwortartig definiert und durch Literaturrecherchen ergänzt wurden. Aus diesen Definitionen wurden Items für jede Zelle der Matrix aus bestehenden Fragebögen ausgewählt bzw. basierend auf aktueller Literatur neu entwickelt. Dabei wurde die Kritik an bestehenden Fragebögen im Rahmen der Beurteilung der Items miteinbezogen^2^. Für die Beantwortung der Items wurde in der ersten Fragebogenversion eine siebenstufige Skala von *sicher falsch *(1) bis *sicher richtig* (7) gewählt.

**Tab. 2:** Darstellung der Struktur der BiPS Matrix mit Beispielitems^a^

Inhaltsbereich	Biologisch	Psychologisch	Sozial	
*Art der Störung*	Z. B. Item 1: „Chronischer Schmerz hat eine Warnfunktion und deutet stets auf eine Schädigung des Körpers hin.“ *(inv.)*	Z. B. Item 8: „Die Stärke des wahrgenommenen Schmerzes wird durch gedankliche Prozesse beeinflusst.“	Z. B. Item 10: „Schmerz wird durch einen spezifischen Gesichtsausdruck kommuniziert.“	*–*
Itemanzahl	5	4	4	14
*Annahmen zur Ursache*	Z. B. Item 14: „Stärkere Verletzungen führen immer zu stärkerem Schmerz.“ *(inv.)*	Z. B. Item 18: „Sie nehmen stärkeren Schmerz wahr, wenn Sie sich über Ihren Schmerz Sorgen machen.“	Z. B. Item 24: „Eine sehr besorgte Reaktion von medizinischem Personal verringert bestehenden Schmerz.“ *(inv.)*	–
Itemanzahl	4	5	5	14
*Konsequenzen*	Z. B. Item 29: „Bei akutem Schmerz können Operationen, die der Behandlung der Schmerzursache dienen, zu einer Linderung der Schmerzen führen.“	Z. B. Item 31: „Dauerhafter oder wiederkehrender Schmerz kann Gefühle von Hilf- und Hoffnungslosigkeit hervorrufen.“	Z. B. Item 35: „Man kann mit dauerhaftem oder wiederkehrendem Schmerz kein sozial aktives Leben führen.“ *(inv.)*	**–**
Itemanzahl	3	4	5	12
*Zeitlicher Krankheitsverlauf*	Z. B. Item 41: „Chronischer Schmerz besteht über den Zeitraum einer möglichen Wundheilung hinaus.“	Z. B. Item 45: „Chronischer Schmerz bedeutet nicht zwangsläufig, dass dieser durchgängig erlebt wird.“	Z. B. Item 48: „Unabhängig von den Menschen, die Sie umgeben, bleibt die Schmerzstärke über den Tag hinweg stabil.“ *(inv.)*	**–**
Itemanzahl	2	5	2	9
*Möglichkeiten der Kontrolle/Behandlung*	Z. B. Item 51: „Einzig und allein Ärztinnen und Ärzte sind in der Lage, dauerhaften oder wiederkehrenden Schmerz zu lindern.“ *(inv.)*	Z. B. Item 53: „Das Anwenden von psychologischen Strategien, wie z. B. Ablenkung, führt zu einer Schmerzverringerung.“	Z. B. Item 61: „Bei dauerhaftem oder wiederkehrendem Schmerz ist es ratsam, soziale Aktivitäten zu reduzieren.“ *(inv.)*	**–**
Itemanzahl	4	6	5	15

*inv.* invertiertes Item

^a^Mögliche Antwortoptionen waren: (1) „Ja, dieses Item ist verständlich und kann unverändert übernommen werden“, (2) „Nein, dieses Item sollte nicht übernommen werden“, (3) „Ja, dieses Item sollte nach sprachlicher Überarbeitung übernommen werden“, (4) „Ja, dieses Item sollte für Kinder und Jugendliche sprachlich verändert übernommen werden“

Eine erste Erprobung einer Vorversion der BiPS Matrix mit insgesamt 72 Items fand an einer Stichprobe von *N* = 47 Erwachsenen aus der Allgemeinbevölkerung (70,21 % weiblich, *M* = 33,55 Jahre), die über persönliche Netzwerke des Forschungsteams nach dem Schneeballprinzip rekrutiert wurden, im Rahmen einer Masterarbeit statt (vgl. Anhang C im Online-Zusatzmaterial, Tab. S1)^3^. Das Ziel war die Berechnung von Itemkennwerten und von psychometrischen Charakteristika (z. B. Reliabilität, Trennschärfe). Die Kürzung des Fragebogens basierte auf inhaltlicher Relevanz, Reliabilität der Items und den Probandenkommentaren: Psychometrisch schwache Items wurden bei einer negativen oder Nulltrennschärfe, einer Trennschärfe von *r*_*it*_ < 0,10 und bei einer Itemvarianz von σ < 1 ausgeschlossen. Psychometrisch schwache Items wurden jedoch behalten und wenn möglich umformuliert, falls kein weiteres Item zum selben Inhalt existierte.

Die internen Konsistenzen lagen für die Inhaltsdimensionen im akzeptablen bis exzellenten Bereich (biologische Skala: *α* = 0,73, psychologische Skala: *α* = 0,92, soziale Skala: *α* = 0,88). Für die Inhaltsbereiche lagen die internen Konsistenzen mit einer Range von 0,72 < *α* < 0,90 ebenso im akzeptablen bis exzellenten Bereich (Art der Störung: *α* = 0,75, Annahmen zur Ursache: *α* = 0,80, Konsequenzen: *α* = 0,72, zeitlicher Krankheitsverlauf: *α* = 0,78, Möglichkeiten der Kontrolle/Behandlung: *α* = 0,90). Hinsichtlich der Validität und inhaltlichen Relevanz der BiPS Matrix erfolgte außerdem die Ermittlung von Zusammenhängen der BiPS Matrix (Gesamtscore) mit schmerzrelevantem Vorwissen und bestehenden akuten und chronischen Schmerzen^4^. Es zeigten sich Zusammenhänge zwischen dem Gesamtscore der BiPS Matrix und dem selbsteingeschätzten Vorwissen der Proband*innen (*r* = 0,33, *p* < 0,01). Weiterhin zeigte die Grundgesamtheit der Proband*innen ohne chronische Schmerzen (69,2 %) einen höheren Gesamtscore in der BiPS Matrix (*t *(45) = 3,34, *p* < 0,01, *d* = 1,01). Basierend auf den Ergebnissen wurde die Vorversion von ursprünglich 72 auf 63 Items reduziert. Diese BiPS Matrix Version wurde für die vorliegende Studie verwendet.

##### Relevanz der Erfassung von Schmerzkonzepten.

In der vorliegenden Studie wurde die wahrgenommene Relevanz der Erfassung von Schmerzkonzepten der Expert*innen über eine fünfstufige Likert-Skala von *überhaupt nicht wichtig (1)* bis *extrem wichtig (5)* separat für die drei Kontexte (1) im Allgemeinen, (2) im klinischen Kontext und (3) im Forschungskontext mit je einem Item erfasst.

##### Relevanz der Inhaltsdimensionen und -bereiche.

Die Einschätzung der Relevanz der Inhaltsdimensionen (biologisch, psychologisch, sozial) wurde auf Itemebene vorgenommen. Weiterhin wurde die Relevanz der Erfassung der fünf Inhaltsbereiche nach dem CSM [9] mit insgesamt fünf Items („Wie wichtig ist es Ihrer Meinung nach, die inhaltliche Dimension „Art der Störung/Annahmen zur Ursache/Konsequenzen der Krankheit für die Betroffenen/Zeitlicher Krankheitsverlauf/Möglichkeiten der Kontrolle und Behandlung“ zu erfassen?“) auf einer fünfstufigen Likert-Skala von *überhaupt nicht wichtig (1)* bis *extrem wichtig (5) *erfasst.

##### Relevanz und Verständlichkeit der Items.

Die Items der BiPS Matrix wurden von den Expert*innen hinsichtlich ihrer Relevanz und Verständlichkeit in der Erfassung von Schmerzkonzepten eingeschätzt („Ist dieses Item wichtig für die Erfassung der Schmerzkonzepte?“). Die zur Verfügung stehenden Antwortoptionen waren: (1) „Ja, dieses Item ist verständlich und kann unverändert übernommen werden“, (2) „Nein, dieses Item sollte nicht übernommen werden“, (3) „Ja, dieses Item sollte nach sprachlicher Überarbeitung übernommen werden“ und (4) „Ja, dieses Item sollte für Kinder und Jugendliche sprachlich verändert übernommen werden“.

##### Sprachliche Überarbeitung der Items aus Expert*innensicht.

Die Expert*innen machten ebenfalls Angaben zu der Notwendigkeit einer sprachlichen Überarbeitung der Items, auch für die Erhebung von Schmerzkonzepten bei Kindern und Jugendlichen.

Die gesamte Umfrage lag in deutscher Sprache vor. Die Bearbeitungszeit betrug zwischen 20 und 30 min.

### Statistische Auswertung

Die Auswertung der Daten erfolgte mit dem Statistikprogramm *IBM SPSS Statistics *(Version 26) und Microsoft Excel (Version 2019). Die soziodemografischen Angaben, Relevanzeinschätzungen und Itembewertungen der Expert*innen wurden deskriptiv mithilfe von Häufigkeitsanalysen ausgewertet.

Für die Auswertung der 271 Expert*innenkommentare zu den Items der Schmerzmatrix wurde auf die qualitative Inhaltsanalyse nach Mayring [17] zurückgegriffen. Dabei wurde eine Kombination aus einem deduktiven und einem induktiven Vorgehen gewählt. Im Rahmen der deduktiven Kategorienbildung wurden à priori folgende Kategorien mit Unterkategorien über einen Codierleitfaden (vgl. Anhang D im Online-Zusatzmaterial) definiert und voneinander abgegrenzt: (1) sprachliche Anmerkungen (a. Formulierung, b. Satzbau und c. Inversion), (2) inhaltliche Anmerkungen (a. allgemeine Verständlichkeit, b. Relevanz, c. Vorwissen erforderlich und d. Kinder/Jugendliche vs. Erwachsene) sowie (3) Bewertung (a. Zustimmung und b. Ablehnung). Die Auswertung erfolgte durch drei Raterinnen (AF, LW, LA), die unabhängig voneinander alle Kommentare in das Kategoriensystem einordneten. Kommentare, die zu keiner Kategorie passten, bildeten eine Restekategorie. Nach dem ersten Rating-Durchgang wurden Differenzen in der Zuordnung der Kommentare analysiert und der Codierleitfaden weiter präzisiert. Aus den Kommentaren der Restekategorie wurde induktiv eine neue Kategorie (4) keine Auswertung auf Itemebene (a. nicht auswertbar, b. nicht relevant für das Forschungsteam und c. allgemeine Hinweise zum Fragebogen) gebildet. Diese Kategorie umfasste jegliche Expert*innenkommentare, aus denen keine Erkenntnisse für die Gestaltung der Items des Fragebogens gewonnen werden konnten. In einem zweiten Durchgang fand die endgültige Zuordnung aller Kommentare zu den Kategorien durch die Raterinnen (AF, LA, LW) statt. Für die Berechnung der Interrater-Reliabilität zwischen den drei Raterinnen wurde Fleiss’ Kappa verwendet. Im Rahmen der qualitativen Inhaltsanalyse [17] ließ sich bei der Zuordnung der Expert*innenkommentare zu den einzelnen Kategorien eine zufriedenstellende Interrater-Reliabilität von Fleiss’ Kappa = 0,628 (*n* = 3) zwischen den drei Raterinnen (AF, LA, LW) aufweisen [15].

## Ergebnisse

### Relevanz der Erfassung von Schmerzkonzepten

**Abb. 1:**
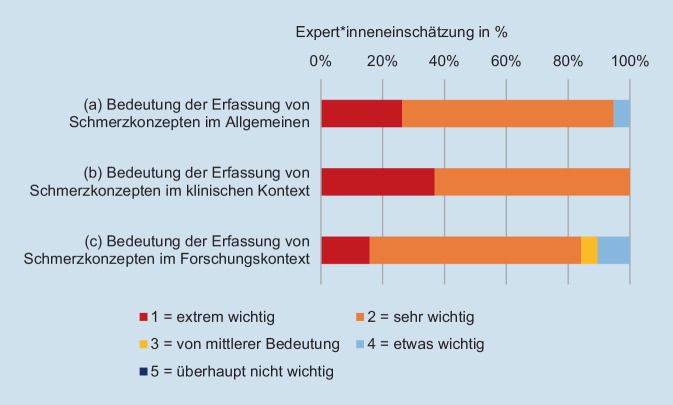
Verteilung der Expert*inneneinschätzungen hinsichtlich der **a** Bedeutung im Allgemeinen, **b** Bedeutung im klinischen Kontext und **c** Bedeutung im Forschungskontext der Erfassung von Schmerzkonzepten bei chronischen Schmerzpatient*innen

In der Befragung gaben 63,2 % der Expert*innen an, Schmerzkonzepte zu erfassen. Ein Großteil der 17 Expert*innen betrachtete die Erfassung von Schmerzkonzepten bei Erwachsenen mit chronischen Schmerzen als *sehr wichtig* (allgemein: 68,4 %, klinischer Kontext: 63,2 %, Forschungskontext: 68,4 %) oder als *extrem wichtig* (allgemein: 26,3 %, klinischer Kontext: 36,8 %, Forschungskontext: 15,8 %; Abb. 1). Keine Person gab an, dass es *überhaupt nicht* wichtig sei, Schmerzkonzepte von chronischen Schmerzpatient*innen zu erfragen. Die Mehrheit der Expert*innen (42,9 %) gab dabei an, auf informelle Methoden (z. B. mündliche Abfrage) zurückzugreifen.

### Relevanz der Inhaltsdimensionen und -bereiche nach dem CSM

**Abb. 2:**
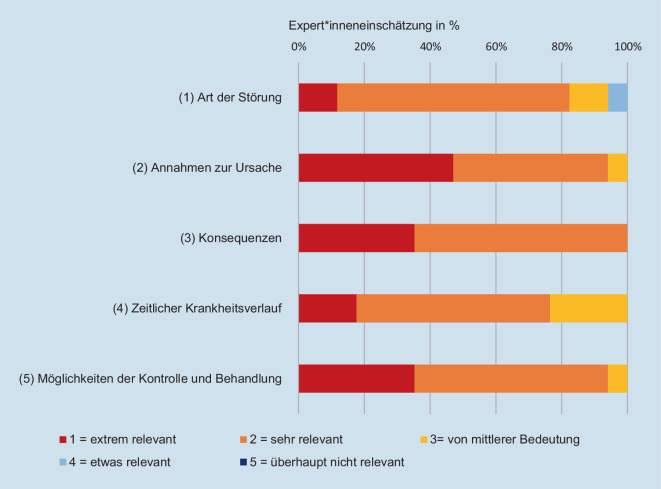
Verteilung der Expert*inneneinschätzungen hinsichtlich der Bedeutung der Erfassung der fünf Inhaltsbereiche von Schmerzkonzepten nach dem CSM [9]

Die Erfassung der fünf Inhaltsbereiche nach dem CSM [9] wurde von mindestens 75 % der 17 Expert*innen als *sehr relevant* oder *extrem relevant* eingeschätzt, geratet auf einer fünfstufigen Likert-Skala von *überhaupt nicht wichtig* (1) bis *extrem wichtig* (5) (Abb. 2). Unterschiede in der Relevanz von Items des biologischen, psychologischen und sozialen Schmerzkonzepts zeigten sich nicht (*p* > 0,05).

### Relevanz (und Verständlichkeit) der Items der BiPS Matrix

Die Einschätzungen der Expert*innen (*n* = 17) zu den 63 Items der biopsychosozialen Schmerzmatrix sind Anhang E (Abb. S1) im Online-Zusatzmaterial zu entnehmen. Hinsichtlich der nachfolgenden Revision der BiPS Matrix wurde im Vorhinein ein Cut-off-Wert festgelegt: Wenn mindestens sechs von 17 Expert*innen (35 %) ein Item ablehnten oder wenn mindestens drei von 17 Expert*innen (17 %) ein Item ablehnten und die zuvor berichtete Trennschärfe (vgl. 5) r_*it*_ < 0,3 betrug, wurde dieses für die revidierte Version der BiPS Matrix nicht übernommen. Somit wurden fünf Items (8 %; Item 7, 10, 24, 39 und 43) nicht übernommen, da diese die zuvor festgelegten Kriterien hinsichtlich Ablehnung erfüllten. Die höchste Ablehnungsquote eines Items beträgt 47 % für Item 39 (*„Infolge von dauerhaftem oder wiederkehrendem Schmerz wird die Beziehung zu dem/der behandelnden Arzt/Ärztin für den/die Betroffene/-n immer wichtiger“.* Sozial, Konsequenzen). Bei 88,9 % der Items gab mindestens ein*e Expert*in an, dass das Item nach sprachlicher Überarbeitung übernommen werden sollte (z. B. Item 37: *„Schmerz kann zur Berufsaufgabe führen“.* Sozial, Konsequenzen). Die sich daraus für die Revision ergebende notwendige sprachliche Überarbeitung wurde zum einen im Rahmen der qualitativen Datenanalyse (s. Punkt 3.4) weiter präzisiert – wenn zusätzlich ein Kommentar vorhanden war – und zum anderen im Anschluss im Forschungsteam diskutiert.

### Qualitative Datenanalyse

Die Zuordnung der insgesamt 271 Expert*innenkommentare zu den 63 Items erfolgte in vier Überkategorien: (1) sprachliche Anmerkungen, (2) inhaltliche Anmerkungen, (3) Bewertung (Zustimmung/Ablehnung), (4) keine Auswertung auf Itemebene (z. B. allgemeine Hinweise zum Fragebogen). Diese vier Überkategorien waren in weitere Subkategorien ausdifferenziert und konkretisiert. Nicht zuzuordnende Kommentare fielen in eine Restkategorie (vgl. Anhang F im Online-Zusatzmaterial, Tab. S2). Dabei wurden von den Expert*innen zu Items, die der Erfassung psychologischer (*n* = 105 Kommentare; 38,75 %) und sozialer Schmerzkonzepte (*n* = 107 Kommentare; 39,48 %) dienen, mehr Kommentare gemacht als zu den Items biologischer (*n* = 59 Kommentare; 21,77 %). Die meisten Kommentare über alle Inhaltsdimensionen und -bereiche hinweg betrafen (1) sprachliche Anmerkungen (*n* = 469 Kommentare; 57,7 %), wobei insbesondere das Sprachniveau bzw. die Itemformulierung angemerkt wurde. Bei den Items der Inhaltsbereiche (2) Annahmen zur Ursache (*n* = 12 Kommentare; 7,02 %) sowie (4) zeitlicher Krankheitsverlauf (*n* = 8 Kommentare; 6,84 %) wurden die meisten invertierten Items kommentiert. Die Unterkategorie „Vorwissen erforderlich“ beinhaltete, dass ein Item nicht ohne gewisse Fertigkeiten und Kenntnisse im Bereich chronischer Schmerzen verstanden wird. Hier zeigt sich, dass lediglich Items in den Inhaltsbereichen (1) Art der Störung (*n* = 10 Kommentare; 4,57 %) und (5) Möglichkeiten der Kontrolle und Behandlung (*n* = 5 Kommentare; 3,62 %) laut Expert*innen Vorwissen benötigen.

### Sprachliche Überarbeitung der Items für Kinder aus Expert*innensicht

Weiterhin ergaben sich Erkenntnisse für die Überarbeitung der Items im Gebrauch mit Kindern und Jugendlichen. Wie Tab. 3 zu entnehmen ist, zielte ein Großteil der Expert*innenkommentare darauf ab, die Itemformulierungen weniger kompliziert (u. a. mit weniger Fachbegriffen) zu gestalten und mit mehr konkreten Beispielen die Lebensrealität der Kinder besser widerzuspiegeln. Die Kommentare ergeben in der hierauf aufbauenden Adaptation der BiPS Matrix für Kinder und Jugendliche wichtige Anhaltspunkte zur Umformulierung der Items.

**Tab. 3:** Items, bei denen mindestens 35 % der Expert*innen eine Umformulierung für die Verwendung mit Kindern und Jugendlichen empfehlen

Itemnummer	Item	Beispiel Expert*innenkommentar
1	Chronischer Schmerz hat eine Warnfunktion und deutet stets auf eine Schädigung des Körpers hin (*invertiert*)	„Warnfunktion sollte erklärt werden“
8	Die Stärke des wahrgenommenen Schmerzes wird durch gedankliche Prozesse beeinflusst	„Mehr umgangssprachlich bleiben, weniger wissenschaftliche Wörter nehmen“
10	Schmerz wird durch einen spezifischen Gesichtsausdruck kommuniziert	„Spezifisch und kommuniziert – bitte keine Fachwörter in einen Kinderfragebogen“
11	Die Schmerzstärke bleibt unverändert, auch wenn Sie sich mit dem Partner oder einem/einer guten Freund/-in streiten (*invertiert*)	„Partner ist für viele Kinder und Jugendliche noch nicht relevant. Eher Familie“
12	Wenn Sie sich verletzen, wird die soziale Umgebung, in der Sie sich befinden, Ihre Schmerzwahrnehmung nicht beeinflussen (*invertiert*)	„Der Satz ist insgesamt zu kompliziert für Kinder und Jugendliche“
13	Die Stärke der Schmerzempfindung ist abhängig von sozialen Faktoren wie der Kultur	„Soziale Faktoren wie Kultur ist v. a. für Kinder wenig greifbar. Beispiele nennen wie (Erziehung, Erfahrungen, das Land in dem du lebst)“
33	Dauerhafter oder wiederkehrender Schmerz führt aufgrund von schmerzbezogener Angst zu einer Schonhaltung und Bewegungsvermeidung, wodurch der Schmerz langfristig reduziert werden kann (*invertiert*)	„Bei uns verstehen die Kinder [die Formulierung] Angst vor Schmerzen besser als schmerzbezogene Ängste“
35	Man kann mit dauerhaftem oder wiederkehrendem Schmerz kein sozial aktives Leben führen (*invertiert*)	„Sozial aktives Leben schwierig zu verstehen, eventuell mit Beispielen (z. B. Freunde treffen, gemeinsam etwas unternehmen)“
37	Schmerz kann zur Berufsaufgabe führen	„Schmerz führt dazu, dass man nicht zur Schule gehen kann“
38	Die soziale Rolle der Betroffenen bleibt trotz dauerhaftem oder wiederkehrendem Schmerz in der Regel unbeeinträchtigt *(invertiert)*	„Anstatt soziale Rolle: sich mit FreundInnen treffen (konkrete Beispiele)“

### Revidierte Fassung der BiPS Matrix für Erwachsene

Aufbauend auf der vorliegenden Studie wurde die BiPS Matrix für Erwachsene weiterentwickelt. Anhand der quantitativen Daten wurden folgende Items aus der BiPS Matrix herausgekürzt (z. B. Item 7, 10, 24, 39 und 43), da diese von mindestens 35 % der Expert*innen abgelehnt wurden bzw. mindestens 17 % der Expert*innen dieses Item ablehnten und die zuvor berichtete Trennschärfe *r*_*it*_ <. 3 betrug. Weiterhin wurde die BiPS Matrix mit vorhandenen Fragebogentools abgeglichen (z. B. [33]), indem die Items eines bereits etablierten Fragebogens inhaltlich passenden Items der BiPS Matrix zugeordnet wurden, um diese weiterzuentwickeln. Redundante, unklare oder zu abstrakte Items wurden auf Basis der Expert*inneneinschätzung identifiziert und innerhalb des Forschungsteams diskutiert. Es wurden außerdem anhand der erhobenen qualitativen Daten weitere Kürzungen des Fragebogens sowie sprachliche Anpassungen über mehrere Rückkopplungsschleifen im Forschungsteam diskutiert und umgesetzt, sodass eine Version mit 40 Items entstand (vgl. Anhang G im Online-Zusatzmaterial, Tab. S3). Diese Version enthält weniger komplexe Sätze zur Steigerung der allgemeinen Verständlichkeit (z. B. Item 8: „Die Stärke des wahrgenommenen Schmerzes wird durch gedankliche Prozesse beeinflusst“ wurde umgewandelt in „Gedanken können die Schmerzstärke beeinflussen“ [jetzt Item 6]). Invertierte Items wurden aufgrund der Gefahr von Deckeneffekten beibehalten, aber auf Basis der Expert*inneneinschätzungen sprachlich einfacher dargestellt (z. B. Item 12: „Wenn Sie sich verletzen, wird die soziale Umgebung, in der Sie sich befinden, Ihre Schmerzwahrnehmung nicht beeinflussen (*inv.*)“ wurde umgewandelt in „Die Schmerzstärke bleibt gleich, unabhängig davon, wo und mit wem man gerade zusammen ist (*inv*.)“ [jetzt Item 9]).

## Diskussion

Die online-basierte Erhebung an 17 Expert*innen ergab, dass die überwiegende Mehrzahl der Expert*innen die Erfassung von Schmerzkonzepten bei chronischen Schmerzpatient*innen im Allgemeinen sowie im klinischen und im Forschungskontext als sehr relevant einschätzte. Die Erfassung der Schmerzkonzepte durch die BiPS Matrix wird durch die Integration der biologischen, psychologischen und sozialen Aspekte sowie der Dimensionen des CSM von den Expert*innen ebenfalls als klinisch relevant eingeschätzt. Unterschiede in der Relevanz von Items des biologischen, psychologischen und sozialen Schmerzkonzepts zeigten sich nicht (*p* > 0,05). Es wurden primär Anmerkungen zur Optimierung der Itemformulierungen gemacht, sowie Vorschläge für eine Anpassung der Items für Kinder. Ein Ausblick für eine adaptierte Version der BiPS Matrix für Erwachsene befindet sich im Online-Zusatzmaterial (Anhang G, Tab. S3).

Zwölf der 17 Expert*innen gaben an, die Schmerzkonzepte ihrer chronischen Schmerzpatient*innen in der ambulanten Behandlung zu erfassen. Die Erfassung von Schmerzkonzepten ist aus Expert*innensicht hoch relevant, auch wenn derzeit kaum standardisierte Messinstrumente existieren [25], die Erfassung also informell und in unterschiedlichster Form erfolgt. Dies belegen auch Pate et al. [25] in ihrer Expert*innenbefragung. Dass aber durch die Etablierung eines standardisierten Instruments, das Schmerzkonzepte inhaltlich breit und mehrdimensional erhebt, schlussendlich die Versorgung von Betroffenen verbessert werden kann, erklärt die durch die Expert*innen eingeschätzte hohe Relevanz der BiPS Matrix. Denn edukative Interventionen, die an vorherrschende Schmerzkonzepte anknüpfen, können Schmerzwissen, Einschränkungen, Katastrophisierung, Vermeidungsverhalten, Bewegung und sogar Inanspruchnahme des Gesundheitssystems verbessern [16].

Die Expert*innenkommentare wurden durch drei unabhängige Rater*innen in die unterschiedlichen Kategorien eingeteilt. Insgesamt ist von einer hohen Übereinstimmung der Expert*innen auszugehen. Bei der vorliegenden Studie hat sich gezeigt, dass die Expert*innen überwiegend sprachliche Anmerkungen zu den Items im Vergleich zu anderen Kommentaren verfasst haben (57,7 %). Im Hinblick auf eine revidierte Version der BiPS Matrix bieten diese konkrete Anhaltspunkte für die sprachliche Optimierung der Items (z. B. „statt ‚stets‘ ‚immer‘ verwenden“). Weiterhin trug die Expertise der Befragten dazu bei, die Itemformulierungen für eine möglichst heterogene Population verständlich zu machen und Fachbegriffe, vor allem für die noch zu konzipierende Kinderversion, größtenteils zu vermeiden (z. B. „Warnfunktion sollte erklärt werden“, insbesondere im Sinne der *„health literacy“ *[29]). Dies zeigt, dass die Expert*innen patientenzentriert arbeiten und somit für eine verständliche Fachsprache sensibilisiert sind. Kritisch waren die Expert*innen hinsichtlich invertiert formulierter Items. Allerdings könnte bei einer Vermeidung von invertierten Items das Risiko von Deckeneffekten und damit wenig aussagekräftigen Ergebnissen bestehen. In jedem Fall sollte bei einer Beibehaltung invertierter Items auf eine möglichst einfache Formulierung geachtet werden, insbesondere bei der zu erstellenden Kinderversion, so die Empfehlung der Expert*innen.

28,54 % der Kommentare bzgl. des Iteminhalts beinhalteten unspezifische Vorschläge für eine Adaptation (z. B. „[Item] erklären“) und es zeigten sich teilweise divergierende Einschätzungen der Expert*innen. Bei der Erstellung einer revidierten Version des Fragebogens erwiesen sich daher die Entscheidung für das Beibehalten oder Verwerfen einzelner Items sowie die Umformulierung als herausfordernd.

### Limitationen

Trotz der innovativen und theoriegeleiteten Konzeption der BiPS Matrix müssen folgende Aspekte kritisch betrachtet werden: Eine Vergrößerung der Stichprobe der befragten Expert*innen sowie der ausgewogene Einbezug internationaler Expert*innen aus unterschiedlichen Fachdisziplinen (Psychologie, Medizin [Ärzte und Pflegepersonal], Physiotherapie, Ergotherapie) würden die Repräsentativität und Validität steigern. Neben einer online-basierten Erhebung der Expert*innen könnten kognitive Interviewverfahren hinzugezogen werden, um die spontane Einschätzung und Kommentierung der Expert*innen direkt zu erfassen.

### Ausblick

Anhand der präsentierten Expert*innenbefragung wurde die BiPS Matrix profunde weiterentwickelt und gekürzt. Eine gekürzte Version wird zur weiteren Erforschung im Anhang zur Verfügung gestellt (vgl. Anhang G im Online-Zusatzmaterial, Tab. S3).

Zukünftige Schritte umfassen u. a. größer angelegte Untersuchungen von Testgütekriterien der BiPS Matrix in klinischen Stichproben, die Untersuchung von Wissensprofilen in diesen Stichproben, um Unterschiede und Ansatzpunkte für eine individualisierte Psychoedukation abzubilden, sowie die Adaptation und Überprüfung der BiPS Matrix an Stichproben mit Kindern.

Nicht zuletzt können in zukünftigen Studien auch Schmerzkonzepte aufseiten der Behandelnden erfasst werden [1]. Eine zentrale Frage der Schmerztherapie betrifft nach wie vor die unzulängliche Behandlung von Betroffenen auch außerhalb der interdisziplinären, stationären Schmerztherapie [28]. Neben den Schmerzkonzepten der Betroffenen bietet die Erfassung der Schmerzkonzepte von Behandelnden die Möglichkeit, Konzepte und Kenntnisse zu diesem prävalenten Störungsbild abzubilden.

## Fazit für die Praxis

- Die Expert*innen bewerten die Erfassung biopsychosozialer Schmerzkonzepte als relevant. Dabei nutzt eine Mehrheit der befragten Expert*innen informelle Erfassungsmethoden, was das Fehlen eines standardisierten Fragebogens hervorhebt.
- Die Expert*innen-Ratings zur neu konzipierten BiPS Matrix, insbesondere die sprachlichen Anmerkungen, ermöglichten die wissenschaftliche Weiterentwicklung dieses multidimensionalen Assessmenttools.
- Die BiPS Matrix ist laut Expert*inneneinschätzung grundsätzlich auch für die Verwendung mit Kindern geeignet. Allerdings ist hier eine weitere, kindgerechte Anpassung erforderlich. Die vorliegende Version stellt eine erste Grundlage für die Weiterentwicklung für Kinder dar.

## Supplementary Information

Fragebögen, Codierleitfaden und vertiefende Zusatzmaterialien

## Footnotes

1 Kloos T (2019) Entwicklung eines Fragebogens zur Erfassung von Schmerzkonzepten. Unveröffentlichte Masterarbeit, Universität Trier

2 Kloos T (2019) Entwicklung eines Fragebogens zur Erfassung von Schmerzkonzepten. Unveröffentlichte Masterarbeit, Universität Trier

3 Kloos T (2019) Entwicklung eines Fragebogens zur Erfassung von Schmerzkonzepten. Unveröffentlichte Masterarbeit, Universität Trier

4 Kloos T (2019) Entwicklung eines Fragebogens zur Erfassung von Schmerzkonzepten. Unveröffentlichte Masterarbeit, Universität Trier

5 Kloos T (2019) Entwicklung eines Fragebogens zur Erfassung von Schmerzkonzepten. Unveröffentlichte Masterarbeit, Universität Trier
