# Supplementary material for: An instrument to assess biopsychosocial pain concepts in adults: Development and evaluation by experts
Source: Schmerz. 2024 Feb 13;39(3):194–203. [Article in German] doi: 10.1007/s00482-024-00793-2 (PMC12098183; doi:10.1007/s00482-024-00793-2)
Supplement: Supplementary file 1 — Fragebögen, Codierleitfaden und vertiefende Zusatzmaterialien [file 482_2024_793_MOESM1_ESM.pdf]

Online-Zusatzmaterial

Inhalt

Anhang A: Soziodemografischer Fragebogen 2
Anhang B: Fragebogen zur Erfassung von biopsychosozialen Schmerzkonzepten 5
Anhang C: Soziodemografische Daten der ersten Erprobung 18
Anhang D: Kodierleitfaden 18
Anhang E: Verteilung der Expert*inneneinschätzungen zu den Items der BiPS Matrix 20
Anhang F: Zuordnung der Expert*innenkommentare 23
Anhang G: Revidierte Version der BiPS Matrix 24

Anhang A: Soziodemografischer Fragebogen

Erste Schritte

Um sicherzustellen, dass Sie die Einschlusskriterien für diese Studie erfüllen, wählen Sie bitte alle auf Sie zutreffenden Punkte aus:

☐ Ich bin Forscher/in mit mindestens zwei Veröffentlichungen zum Thema chronische Schmerzen.

☐ Ich bin arbeite seit mindestens zwei Jahren im Bereich chronische Schmerzen.

☐ Weder noch

Soziodemographische Angaben

Zuerst einmal würden wir uns über ein paar kurze Details über Sie freuen…

1. *Wie alt sind Sie?*
   Bitte geben Sie Ihr Alter in ganzen Jahren an: _________

2. *Bitte geben Sie ihr Geschlecht an*:
   ☐ weiblich
   ☐ männlich
   ☐ divers
   ☐ Keine Angabe

3. *Welche Staatsangehörigkeit besitzen Sie?*

   ____

4. *Was ist Ihr höchster formeller Bildungsabschluss?*
   ☐ Berufsausbildung
   ☐ Fachhochschul-/Universitätsabschluss
   ☐ Promotion
   ☐ Habilitation

5. *Besitzen Sie eine oder mehrere Zusatzqualifikationen im Bereich chronische Schmerzen?*
   ☐ Nein
   ☐ ja, welche: ____________

6. *Was beschreibt am besten Ihre Rolle im Bereich chronische Schmerzen? (Alle zutreffenden auswählen)*

   ☐ Kliniker/in
   ☐ Forscher/in
   ☐ Andere:________________

7. *Was ist Ihre Fachdisziplin?*

   ☐ Medizin: Fachrichtung: __________________
   ☐ Psychologie
   ☐ Physiotherapie

☐ Krankenpflege
☐ Ergotherapie
☐ Andere: ______________

8. Mit welcher Patient/innengruppe arbeiten Sie vorwiegend?
(Alle zutreffenden auswählen)

☐ Kinder
☐ Jugendliche
☐ Erwachsene

Vorerfahrungen in der Behandlung von chronischen Schmerzen

Im Folgenden möchten wir gerne mehr über Ihre Vorerfahrungen in der Behandlung von chronischen Schmerzpatient/innen erfahren.

9. *Arbeiten Sie schwerpunktmäßig mit Patient/innen mit chronischen Schmerzen*?
☐ Nein
☐ Ja

10. *Wie lange arbeiten Sie schon im Bereich chronische Schmerzen?*

☐ überhaupt nicht
☐ < 1 Jahr
☐ 1-5 Jahre
☐ 6-10 Jahre
☐ 11+ Jahre

11. *Wie viele Ihrer Patient/innen haben regelmäßig wiederkehrende/chronische Schmerzen?*
☐ 5 – 10 %
☐ 10 – 20%
☐ 20 – 40 %
☐ 50%
☐ > 50 %

12. Primäre chronische Schmerzen werden als Schmerzen in einer oder mehreren Körperregionen, die über einen definierten Zeitraum von 3 Monaten (bei Kindern und Jugendlichen) bzw. 6 Monaten (bei Erwachsenen) anhalten, definiert.
Wie viele Ihrer Patient/innen haben primäre chronische Schmerzen?
☐ 5 – 10 %
☐ 10 – 20%
☐ 20 – 40 %
☐ 50%
☐ > 50 %

13. *Wie viele Schmerzpatient/innen behandeln Sie in einer durchschnittlichen Woche?*
☐ 0
☐ 1-5
☐ 6-10
☐ 11-20
☐ 20+

14. *Erfassen Sie derzeit das Schmerzkonzept, d.h. das Wissen Ihrer Patient/innen über Schmerzen, in der ambulanten Behandlung?*
    - ☐ Nein
    - ☐ Ja

15. *Falls ja, wie erfassen Sie das Schmerzkonzept von Patient/innen in der klinischen Praxis?*
    - ☐ Informell auf der Grundlage klinischer Wahrnehmungen
    - ☐ Verwendung des überarbeiteten Neurophysiology of Pain Questionnaire (rNPQ)
    - ☐ Verwendung einer modifizierten Version des rNPQ
    - ☐ Mit unserem eigenen Fragebogen
    - ☐ Anderes und zwar: ____

16. *Jede/r Behandler/in hat Patientengruppen, die sie/er besonders gerne bzw. ungerne behandelt. Wie gerne behandeln Sie Patient/innen mit chronischen Schmerzen?*
    - ☐ sehr gerne
    - ☐ gerne
    - ☐ neutral
    - ☐ ungerne
    - ☐ sehr ungerne

Anhang B: Fragebogen zur Erfassung von biopsychosozialen Schmerzkonzepten

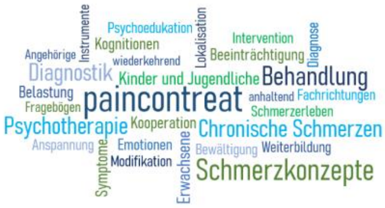

Fragebogen zur Erfassung des bio-psycho-sozialen Schmerzkonzeptes

Ein Schmerzkonzept ist definiert, als das Verständnis, welches eine Person davon hat, was Schmerz eigentlich ist, welche Funktionen Schmerz hat, und welche Prozesse zu dessen Entstehung, Aufrechterhaltung und Behandlung beitragen. Da sowohl biologische, psychologische als auch soziale Prozesse relevant sind, wurde mit diesem Fragebogen eine Matrix entwickelt, in der spaltenweise biologische, psychologische und soziale Aspekte des Schmerzes berücksichtig wurden. Zeilenweise wurden fünf Inhaltsdimensionen – 1) Art der Störung, 2) Annahmen zur Ursache, 3) Konsequenzen des Schmerzes für Betroffene, 4) Zeitlicher Krankheitsverlauf, 5) Möglichkeiten der Kontrolle und Behandlung – erhoben. So entstand ein Fragebogen mit einer 3 (Aspekte) * 5 (Dimensionen) Matrix, zur Erfassung des bio-psycho-sozialen Schmerzkonzeptes. In der aktuellen Version umfasst die Matrix 63 Items.

Als Expert/in im Bereich des Schmerzes bitten wir Sie im Folgenden, für die aufgeführten Items einzuschätzen, für wie wichtig Sie diese erachten, ob Sie die Items als verständlich einschätzen, oder ob Sie Vorschläge für mögliche Umformulierungen haben.

Die folgende Erhebung ist so strukturiert, dass wir Ihnen zunächst allgemeine Fragen zum Schmerzkonzept unterbreiten. Danach erfolgt Ihre Einschätzung zur Wichtigkeit und Verständlichkeit der Items zur Erfassung des bio-psycho-sozialen Schmerzkonzeptes.

Allgemeine Fragen

1. *Wie wichtig ist es Ihrer Meinung nach, das Schmerzkonzept von Patient/innen mit chronischen Schmerzen (Kinder, Jugendliche, Erwachsene) zu erfassen?*

Überhaupt nicht wichtig	Etwas wichtig	Von mittlerer Bedeutung	Sehr wichtig	Extrem wichtig
☐	☐	☐	☐	☐

2. *Wie wichtig halten Sie die Erfassung des Schmerzkonzeptes von Patient/innen mit chronischen Schmerzen im klinischen Kontext und/oder im Forschungskontext?*

Erfassung des Schmerzkonzeptes

	Überhaupt nicht wichtig	Etwas wichtig	Von mittlerer Bedeutung	Sehr wichtig	Extrem wichtig	Anmerkung:
Klinischer Kontext	☐	☐	☐	☐	☐	
Forschungs-kontext	☐	☐	☐	☐	☐	

Ihre Einschätzung zur Wichtigkeit und Verständlichkeit der Items zur Erfassung des bio-psycho-sozialen Schmerzkonzeptes

1. Art der Störung

Wie wichtig ist es Ihrer Meinung nach, die inhaltliche Dimension „Art der Störung", also das Verständnis von Betroffenen über die Art der Störung, zu erfassen?

Überhaupt nicht wichtig	Etwas wichtig	Von mittlerer Bedeutung	Sehr wichtig	Extrem wichtig
☐	☐	☐	☐	☐

Ist **dieses Item** wichtig für die Erfassung der Schmerzkonzepte?

	Ja, dieses Item ist verständlich und kann unverändert übernommen werden	Nein, dieses Item sollte nicht übernommen werden	Ja, dieses Item sollte nach sprachlicher Überar-beitung übernommen werden	Ja, dieses Item sollte für Kinder und Jugendliche sprachlich verändert übernommen werden	Anmerkung bzw. Vorschlag für mögliche Umformulierung des Items
**Biologisch**					
1. Chronischer Schmerz hat eine Warnfunktion und deutet stets auf eine Schädigung des Körpers hin. *(inv.)*	☐	☐	☐	☐	

2. Akuter Schmerz hat eine Schutzfunktion für den Körper	☐	☐	☐	☐	
3. Schmerz tritt immer auf, wenn Sie sich verletzen. *(inv.)*	☐	☐	☐	☐	
4. Spezielle Nervenzellen im Rückenmark übertragen Warnsignale an Ihr Gehirn.	☐	☐	☐	☐	
5. Wenn Sie verletzt sind, übertragen spezielle Rezeptoren ein Warnsignal an Ihr Rückenmark.	☐	☐	☐	☐	
**Psychologisch**					
6. Schmerz entsteht nicht im Gehirn, sondern kommt durch die Nerven in das Gehirn hinein. *(inv.)*	☐	☐	☐	☐	
7. Schmerz ist ein objektives und unangenehmes Sinnes- oder Gefühlserlebnis. *(inv.)*	☐	☐	☐	☐	
8. Die Stärke des wahrgenommenen Schmerzes wird durch gedankliche Prozesse beeinflusst.	☐	☐	☐	☐	
9. Die Schmerzstärke ist unabhängig davon, was man über Schmerzen weiß. *(inv.)*	☐	☐	☐	☐	
**Sozial**					
10. Schmerz wird durch einen spezifischen Gesichtsausdruck kommuniziert.	☐	☐	☐	☐	
11. Die Schmerzstärke bleibt unverändert, auch wenn Sie sich mit dem Partner oder einem/einer guten Freund/-in streiten. *(inv.)*	☐	☐	☐	☐	
12. Wenn Sie sich verletzen, wird die soziale Umgebung, in					

der Sie sich befinden, Ihre Schmerzwahr-nehmung nicht beeinflussen. *(inv.)*	☐	☐	☐	☐	
13. Die Stärke der Schmerzempfindung ist abhängig von sozialen Faktoren wie der Kultur	☐	☐	☐	☐	

2. Annahmen zur Ursache

Wie wichtig ist es Ihrer Meinung nach, die „Annahmen zur Ursache" zu erfassen?

Überhaupt nicht wichtig	Etwas wichtig	Von mittlerer Bedeutung	Sehr wichtig	Extrem wichtig
☐	☐	☐	☐	☐

Im Folgenden bitten wir Sie, für die aufgeführten Items einzuschätzen, ob Sie diese für die Erfassung von Schmerzkonzepten wichtig finden und ob Sie Vorschläge für mögliche Umformulierungen, z.B. für die Altersgruppe Kinder, haben.

Ist **dieses Item** wichtig für die Erfassung der Schmerzkonzepte?

	Ja, dieses Item ist verständlich und kann unverändert übernommen werden	Nein, dieses Item sollte nicht übernommen werden	Ja, dieses Item sollte nach sprachlicher Überar-beitung übernommen werden	Ja, dieses Item sollte für Kinder und Jugendliche sprachlich verändert übernommen werden	Anmerkung bzw. Vorschlag für mögliche Umformu-lierung des Items
**Biologisch**					
14. Stärkere Verletzungen führen immer zu stärkerem Schmerz. *(inv.)*	☐	☐	☐	☐	
15. Chronischer Schmerz bedeutet, dass eine Verletzung nicht richtig geheilt ist. *(inv.)*	☐	☐	☐	☐	
16. Schmerz tritt nur dann auf, wenn Sie sich verletzt haben oder die Gefahr einer Verletzung besteht. *(inv.)*	☐	☐	☐	☐	
17. Durch fehlende körperliche Betätigung oder das Vermeiden	☐	☐	☐	☐	

bestimmter Bewegungen (z. B. aus Angst vor Schmerzen) werden Muskeln abgebaut und es kommt zu einer verstärkten Schmerzwahr-nehmung.					
**Psychologisch**					
18. Sie nehmen stärkeren Schmerz wahr, wenn Sie sich über Ihren Schmerz Sorgen machen.	☐	☐	☐	☐	
19. Schon- und Vermeidungsverhalten bei chronischem Schmerz führen dazu, dass schmerzbezogene Ängste abnehmen und damit weniger starker Schmerz wahrgenommen wird. *(inv.)*	☐	☐	☐	☐	
20. Belastende oder traurige Gedanken haben keinen Einfluss auf die Schmerzstärke. *(inv.)*	☐	☐	☐	☐	
21. Unterdrückter Ärger kann dazu führen, dass intensiverer Schmerz wahrgenommen wird.	☐	☐	☐	☐	
22. Die Art, wie Sie über Ihren Schmerz nachdenken, lässt Ihren Schmerz unbeeinflusst. *(inv.)*	☐	☐	☐	☐	
**Sozial**					
23. Die Schmerz-wahrnehmung kann durch die Art und Weise, wie Ärztinnen und Ärzte über ein Schmerzproblem aufklären, verändert werden.	☐	☐	☐	☐	
24. Eine sehr besorgte Reaktion von medizinischem Personal verringert	☐	☐	☐	☐	

bestehenden Schmerz. *(inv.)*					
25. Permanente Aufmerksamkeit von nahen Bezugspersonen auf andauernden Schmerz kann zu stärkerem Schmerz führen.	☐	☐	☐	☐	
26. Ein stark fürsorgliches und besorgtes Verhalten von nahen Bezugspersonen trägt zu einer geringeren Schmerzwahr-nehmung bei. *(inv.)*	☐	☐	☐	☐	
27. Ängstliche Reaktionen von nahen Bezugspersonen auf eine Verletzung können zu stärkerem Schmerz führen.	☐	☐	☐	☐	

3. Konsequenzen des Schmerzes für Betroffene

Wie wichtig ist es Ihrer Meinung nach, die „Konsequenzen des Schmerzes für Betroffene“ zu erfassen?

Überhaupt nicht wichtig	Etwas wichtig	Von mittlerer Bedeutung	Sehr wichtig	Extrem wichtig
☐	☐	☐	☐	☐

Im Folgenden bitten wir Sie, für die aufgeführten Items einzuschätzen, ob Sie diese für die Erfassung von Schmerzkonzepten wichtig finden und ob Sie Vorschläge für mögliche Umformulierungen, z.B. für die Altersgruppe Kinder, haben.

Ist **dieses Item** wichtig für die Erfassung der Schmerzkonzepte?

	Ja, dieses Item ist verständlich und kann unverändert übernommen werden	Nein, dieses Item sollte nicht übernommen werden	Ja, dieses Item sollte nach sprachlicher Überarbeitung übernommen werden	Ja, dieses Item sollte für Kinder und Jugendliche sprachlich verändert übernommen werden	Anmerkung bzw. Vorschlag für mögliche Umformu-lierung des Items
**Biologisch**					
28. Der Übergebrauch von Schmerzmedikamenten zur Linderung von Schmerzen kann dazu führen, dass Schmerzen langfristig bestehen bleiben.	☐	☐	☐	☐	
29. Bei akutem Schmerz können Operationen, die der Behandlung der Schmerzursache dienen, zu einer Linderung der Schmerzen führen.‘	☐	☐	☐	☐	
30. Bei dauerhaftem oder wiederkehrendem Schmerz ist der erste Ansprechpartner ein/-e Arzt/Ärztin im Krankenhaus. *(inv.)*	☐	☐	☐	☐	
**Psychologisch**					
31. Dauerhafter oder wiederkehrender Schmerz kann Gefühle von Hilf- und Hoffnungs-losigkeit hervorrufen.	☐	☐	☐	☐	
32. Infolge von dauerhaftem oder wiederkehrendem Schmerz kommt es oft zu Beeinträchti-gungen im Tagesablauf der Betroffenen.	☐	☐	☐	☐	

33. Dauerhafter oder wiederkehrender Schmerz führt aufgrund von schmerzbezo-gener Angst zu einer Schonhaltung und Bewegungsvermeidung, wodurch der Schmerz langfristig reduziert werden kann. *(inv.)*	☐	☐	☐	☐	
34. Schmerzbedingte Einschränkungen im Leben sind unabhängig von der Einstellung, die Sie zu den Schmerzen haben. *(inv.)*	☐	☐	☐	☐	
**Sozial**					
35. Man kann mit dauerhaftem oder wiederkehrendem Schmerz kein sozial aktives Leben führen. *(inv.)*	☐	☐	☐	☐	
36. Oft führt andauernder Schmerz dazu, dass man sich von Freundinnen und Freunden sowie sozialen Aktivitäten zurückzieht.	☐	☐	☐	☐	
37. Schmerz kann zur Berufsaufgabe führen.	☐	☐	☐	☐	
38. Die soziale Rolle der Betroffenen bleibt trotz dauerhaftem oder wiederkehrendem Schmerz in der Regel unbeeinträchtigt. *(inv.)*	☐	☐	☐	☐	
39. Infolge von dauerhaftem oder wiederkehrendem Schmerz wird die Beziehung zu dem/der behandelnden	☐	☐	☐	☐	

Arzt/Ärztin für den/die Betroffene/-n immer wichtiger.					

4. Zeitlicher *Krankheitsverlauf*

Wie wichtig ist es Ihrer Meinung nach, den „Zeitlichen Krankheitsverlauf“ zu erfassen?

Überhaupt nicht wichtig	Etwas wichtig	Von mittlerer Bedeutung	Sehr wichtig	Extrem wichtig
☐	☐	☐	☐	☐

Im Folgenden bitten wir Sie, für die aufgeführten Items einzuschätzen, ob Sie diese für die Erfassung von Schmerzkonzepten wichtig finden und ob Sie Vorschläge für mögliche Umformulierungen, z.B. für die Altersgruppe Kinder, haben.

Ist **dieses Item** wichtig für die Erfassung der Schmerzkonzepte?

	Ja, dieses Item ist verständlich und kann unverändert übernommen werden	Nein, dieses Item sollte nicht übernommen werden	Ja, dieses Item sollte nach sprachlicher Überarbeitung übernommen werden	Ja, dieses Item sollte für Kinder und Jugendliche sprachlich verändert übernommen werden	Anmerkung bzw. Vorschlag für mögliche Umformu-lierung des Items
**Biologisch**					
40. Akuter Schmerz ist normalerweise an erkennbare Auslöser gekoppelt. Sobald der Heilungsprozess abgeschlossen ist, klingen auch die Schmerzen ab.	☐	☐	☐	☐	
41. Chronischer Schmerz besteht über den Zeitraum einer möglichen Wundheilung hinaus.	☐	☐	☐	☐	
**Psychologisch**					
42. In einem Moment, in dem Sie glücklich sind, nehmen Sie Schmerz gleich stark wahr wie in einem	☐	☐	☐	☐	

Moment, in dem Sie traurig sind. *(inv.)*					
43. Die Schmerzent-wicklung ist unabhängig davon, ob man befürchtet, dass man dauerhaften oder wieder-kehrenden Schmerz nie wieder los wird, sobald man ihn einmal hat. *(inv.)*	☐	☐	☐	☐	
44. Unabhängig davon, ob man den Schmerz gerade als bedrohlich wahrnimmt oder nicht, bleibt die Schmerzstärke über den Tag hinweg stabil. *(inv.)*	☐	☐	☐	☐	
45. Chronischer Schmerz bedeutet nicht zwangsläufig, dass dieser durchgängig erlebt wird.	☐	☐	☐	☐	
46. Wenn Sie unter chronischem Schmerz leiden, können Sie keine schmerzfreien Momente mehr erleben. *(inv.)*	☐	☐	☐	☐	
**Sozial**					
47. In Abhängigkeit davon, ob Sie gerade mit einem guten Freund zusammen sind oder ein schwieriges Gespräch mit einer unangenehmen Person führen müssen, schwankt die Schmerzstärke über den Tag.	☐	☐	☐	☐	

48. Unabhängig von den Menschen, die Sie umgeben, bleibt die Schmerzstärke über den Tag hinweg stabil. *(inv.)*	☐	☐	☐	☐	

5. Möglichkeiten der Kontrolle und Behandlung

Wie wichtig ist es Ihrer Meinung nach, den „Kontrolle und Behandlung" zu erfassen?

Überhaupt nicht wichtig	Etwas wichtig	Von mittlerer Bedeutung	Sehr wichtig	Extrem wichtig
☐	☐	☐	☐	☐

Im Folgenden bitten wir Sie, für die aufgeführten Items einzuschätzen, ob Sie diese für die Erfassung von Schmerzkonzepten wichtig finden und ob Sie Vorschläge für mögliche Umformulierungen, z.B. für die Altersgruppe Kinder, haben.

Ist **dieses Item** wichtig für die Erfassung der Schmerzkonzepte?

	Ja, dieses Item ist verständlich und kann unverändert übernommen werden	Nein, dieses Item sollte nicht übernommen werden	Ja, dieses Item sollte nach sprachlicher Überarbeitung übernommen werden	Ja, dieses Item sollte für Kinder und Jugendliche sprachlich verändert übernommen werden	Anmerkung bzw. Vorschlag für mögliche Umformu-lierung des Items
**Biologisch**					
49. Um akuten Schmerz angemessen zu behandeln, bedarf es stets einer entsprechenden Schmerz-medikation. *(inv.)*	☐	☐	☐	☐	
50. Die einzige Möglichkeit, mit dauerhaftem oder wiederkehrendem Schmerz umzugehen, ist, Schmerz-medikamente einzunehmen, sobald Schmerz auftritt. *(inv.)*	☐	☐	☐	☐	

51. Einzig und allein Ärztinnen und Ärzte sind in der Lage, dauerhaften oder wiederkehrenden Schmerz zu lindern. *(inv.)*	☐	☐	☐	☐	
52. Körperliche Übungen tragen dazu bei, dauerhaften oder wiederkehrenden Schmerz zu lindern.	☐	☐	☐	☐	
**Psychologisch**					
53. Das Anwenden von psychologischen Strategien, wie z. B. Ablenkung, führt zu einer Schmerzverringerung.	☐	☐	☐	☐	
54. Das Hören von angenehmer Musik kann zwar dazu führen, dass man sich entspannt, beeinflusst jedoch nicht die Schmerzwahrnehmung. *(inv.)*	☐	☐	☐	☐	
55. Wenn man sich entspannt, ist es einfacher, mit Schmerz umzugehen.	☐	☐	☐	☐	
56. Psychologische Schmerzbewältigungsstrategien können zwar hilfreich sein, sind aber kein wesentlicher Bestandteil in der Behandlung von chronischen Schmerzen. *(inv.)*	☐	☐	☐	☐	
57. Wenn der Zahnarzt einem die Möglichkeit bietet, die Behandlung durch ein Handzeichen zu unterbrechen, wird die Behandlung als weniger schmerz-haft empfunden.	☐	☐	☐	☐	

58. Schmerz ist besser zu dulden, wenn man generell davon überzeugt ist, schwierige Situationen aus eigener Kraft bewältigen zu können.	☐	☐	☐	☐	
**Sozial**					
59. Schmerz kann verringert werden, indem man sich mit Freundinnen und Freunden trifft.	☐	☐	☐	☐	
60. Regelmäßig sozialen Aktivitäten nachzukommen, wie z. B. mit Freundinnen und Freunden ins Kino zu gehen, führt zwar zu mehr Spaß im Leben, hat aber keinen Einfluss auf die bestehende Schmerzstärke. *(inv.)*	☐	☐	☐	☐	
61. Bei dauerhaftem oder wiederkehrendem Schmerz ist es ratsam, soziale Aktivitäten zu reduzieren. *(inv.)*	☐	☐	☐	☐	
62. Nahe Bezugspersonen (z.B. Eltern) haben keinen Einfluss auf die Wahrnehmung von anhaltendem Schmerz. *(inv.)*	☐	☐	☐	☐	
63. Das Behandlungsteam kann durch seinen Umgang mit dem/der Betroffenen helfen, dass weniger starker Schmerz wahrgenommen wird.	☐	☐	☐	☐	

Anhang C: Soziodemografische Daten der ersten Erprobung

Tabelle S1

Soziodemografische Daten der ersten Erprobung der BiPS Matrix (N = 47)

**Variable**	***N = 47***	**%**
Geschlecht		
Weiblich	33	70,21
Männlich	14	29,79
Höchster formeller Schulabschluss		
Hauptschulabschluss	1	2,1
Realschulabschluss	2	4,3
(Fach-)abitur	44	93,6
Art der Beschäftigung [a]		
Vollzeit	17	36,2
Teilzeit / Minijob	6	12,8
Immatrikulation	21	44,7
Nicht berufstätig	14	29,8
Pension	4	8,5
Höchster formeller Bildungsabschluss		
Berufsausbildung	5	10,6
Bachelorabschluss	14	29,8
Masterabschluss/Diplom	18	38,3
Promotion	10	21,3
Selbsteingeschätztes, schmerzbezogenes Vorwissen		
Sehr niedrig	2	4,3
Niedrig	11	23,4
Mittel	22	46,7
Hoch	10	21,3
Sehr hoch	2	4,3

[a] Mehrfachantwort möglich.

Anhang D: Kodierleitfaden

Kategorie	Definition	Ankerbeispiele	Kodierregeln
**K1: Sprachliche Anmerkungen**	Alle Anmerkungen, die die Sprache des Items betreffen		
**K1a: Formulierung**	Ausdruck des Texts: verwendete Begriffe, Anrede/Person, Genauigkeit/Eindeutigkeit, Sprachniveau, Wortschatz	„statt stets immer da sprachlich einfacher“ (zu Item 1)	Alle Anmerkungen, die auf die Verwendung bestimmter Begriffe, das Niveau des Sprachstils sowie die Komplexität in der sprachlichen Formulierung der Items eingehen. Keine Anmerkungen, die die inhaltliche Verständlichkeit des Items betreffen.
**K1b: Satzbau**	Konstruktion des Satzes: Wortstellung, Konjugationen, Haupt- und Nebensatz betreffend	„Ich finde den Satzaufbau zu kompliziert. Es sollte einfacher und ohne eingeschobenen Satz formuliert werden.“ (zu Item 12)	Alle Anmerkungen, die sich auf die Gestalt eines Satzes, die Anordnung von Wörtern und Satzgliedern sowie die Verwendung von Haupt- und Nebensätzen beziehen.
**K1c: Inversion**	Umkehr, Umdrehung der Bedeutung	„lieber nicht invers: Belastende oder traurige Gedanken haben Einfluss auf die Schmerzstärke“ (zu Item 20)	Alle Anmerkungen, die sich auf die Inversion mancher Items im Fragebogen beziehen (invertierte Items).
**K2: Inhaltliche Anmerkungen**	Alle Anmerkungen, die die Bedeutung des Items betreffen		
**K2a: Allgemeine Verständlichkeit**	Klarheit, Deutlichkeit, Einfachheit des Items der Inhalt kann vom Leser so verstanden werden, wie der Schreiber es meint	„erneut: ...kein sozial aktives Leben führen- was soll das sein? Konkret wäre besser zu beantworten.“ (zu Item 35)	Alle Anmerkungen, die sich auf die Deutlichkeit und Einfachheit in der Verständlichkeit des Inhalts der Items beziehen. Keine Anmerkungen, die das Sprachniveau und die Komplexität sprachlicher Ausdrücke betreffen.
**K2b: Relevanz**	Wichtigkeit des Items für die Subdomäne oder den Fragebogen	„Ist sehr ähnlich zu bereits formulierten Items.“ (zu Item 62)	Alle Anmerkungen, die auf die Wichtigkeit sowie Redundanz von Items eingehen.
**K2c: Vorwissen erforderlich**	Item wird nicht ohne gewisse Fertigkeiten und Kenntnisse in einem bestimmten Gegenstandsbereich (chronische Schmerzen) verstanden	„Was sind denn psychologische Schmerzbewältigungsstrategien??? Viele Patienten haben davon noch nie gehört...“ (zu Item 56)	Alle Anmerkungen, die sich darauf beziehen, dass die Items aufgrund fehlenden Wissens nicht verstanden werden könnten. Keine Anmerkungen, die ein Nicht-Verstehen, geschuldet durch inhaltliche und sprachliche Komplexität, betreffen.

**K2d: Kinder/ Jugendliche vs. Erwachsene**	Änderung jeglicher Art für Kinder und Jugendliche	„Schmerz wahrnehmen ist manchmal etwas schwierig für Jugendliche und Kinder. Leichter tun sie sich mit ist der Schmerz gleich stark - auch wenn das eine etwas andere Aussage ist.“ (zu Item 42)	Alle Anmerkungen, die auf den Unterschied zwischen erwachsenen Personen und Kindern/Jugendlichen bzgl. der inhaltlichen und sprachlichen Verständlichkeit der Items eingehen.
**K3: Bewertung**	Alle Anmerkungen, die eine Wertung des Items enthalten		
**K3a: Zustimmung**	Ausdruck von Bejahung und Einverständnis mit dem Item	„dieses ist wirklich gut!“ (zu Item 46)	Alle Anmerkungen, die einfache Bejahung und Einverständnis mit dem Item zeigen.
**K3b: Ablehnung**	Ausdruck von Zurückweisung des Items	„Kein gutes Item!“ (zu Item 37)	Alle Anmerkungen, die deutliche Zurückweisung des Items zeigen.
**K4: Keine Auswertung auf Itemebene**	Alle Anmerkungen, bei denen keine Auswertung auf Itemebene stattfindet		
**K4a: Nicht auswertbar**	Nicht zum Auswerten geeignet; es können keine Ergebnisse gewonnen werden	„Ich verstehe das Item nicht. Eigentlich ist doch akuter Schmerz für die Warnfunktion zuständig.“ (zu Item 1) „s.o.“ (zu Item 27)	Alle Anmerkungen, die Verständnisfragen der Expert/innen bezüglich der Items (insbesondere bei invertierten Items) sowie unklare Zusammenhängen beinhalten. Keine Anmerkungen, die die Verständlichkeit der Items durch die Patient/innen betreffen.
**K4b: Nicht relevant für das Forschungsteam**	Im Zusammenhang mit der Auswertung nicht bedeutsam	„das ist doch wohl invers gemeint, oder?“ (zu Item 26)	Alle Anmerkungen, die an der eigentlichen Intention der Expertenbefragung vorbeigehen und nicht die Erwartungen des Forschungsteams treffen. Alle Anmerkungen, die auf Fehler bzgl. der Items hinweisen, die dem Forschungsteam bereits aufgefallen sind.
**K4c: Allgemeine Hinweise zum Fragebogen**	Rat; Tipp; Bemerkung, die in Richtung des Fragebogens zielt und diesbezüglich etwas nahelegt	„Dopplung mit anderen Fragen, zudem viel zu viele Fragen!, reduzieren sie die Fragen insgesamt auf maximal 20!“ (zu Item 62)	Alle Anmerkungen, die auf die allgemeine Konstruktion des Fragebogens eingehen und Tipps diesbezüglich geben (Anzahl der Items etc.).
**K5: Restkategorie**	Alle Anmerkungen, die keiner anderen Kategorie zugeordnet werden können		

Anhang E: Verteilung der Expert*inneneinschätzungen zu den Items der BiPS Matrix

*Abbildung S1.*Verteilung der Expert*inneneinschätzungen zu den Items der BiPS Matrix (für die Struktur der BiPS Matrix s. Tab. 2)

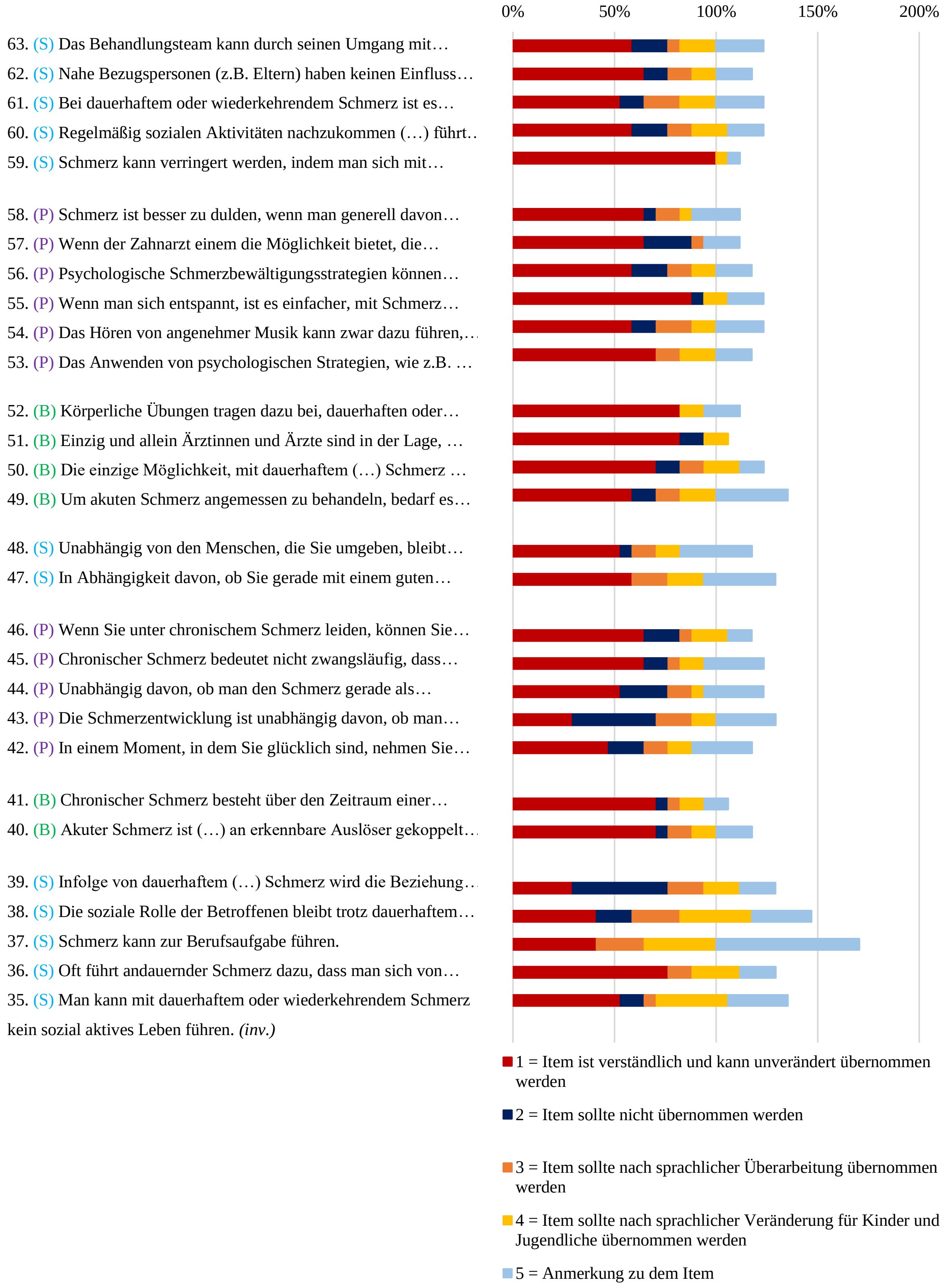

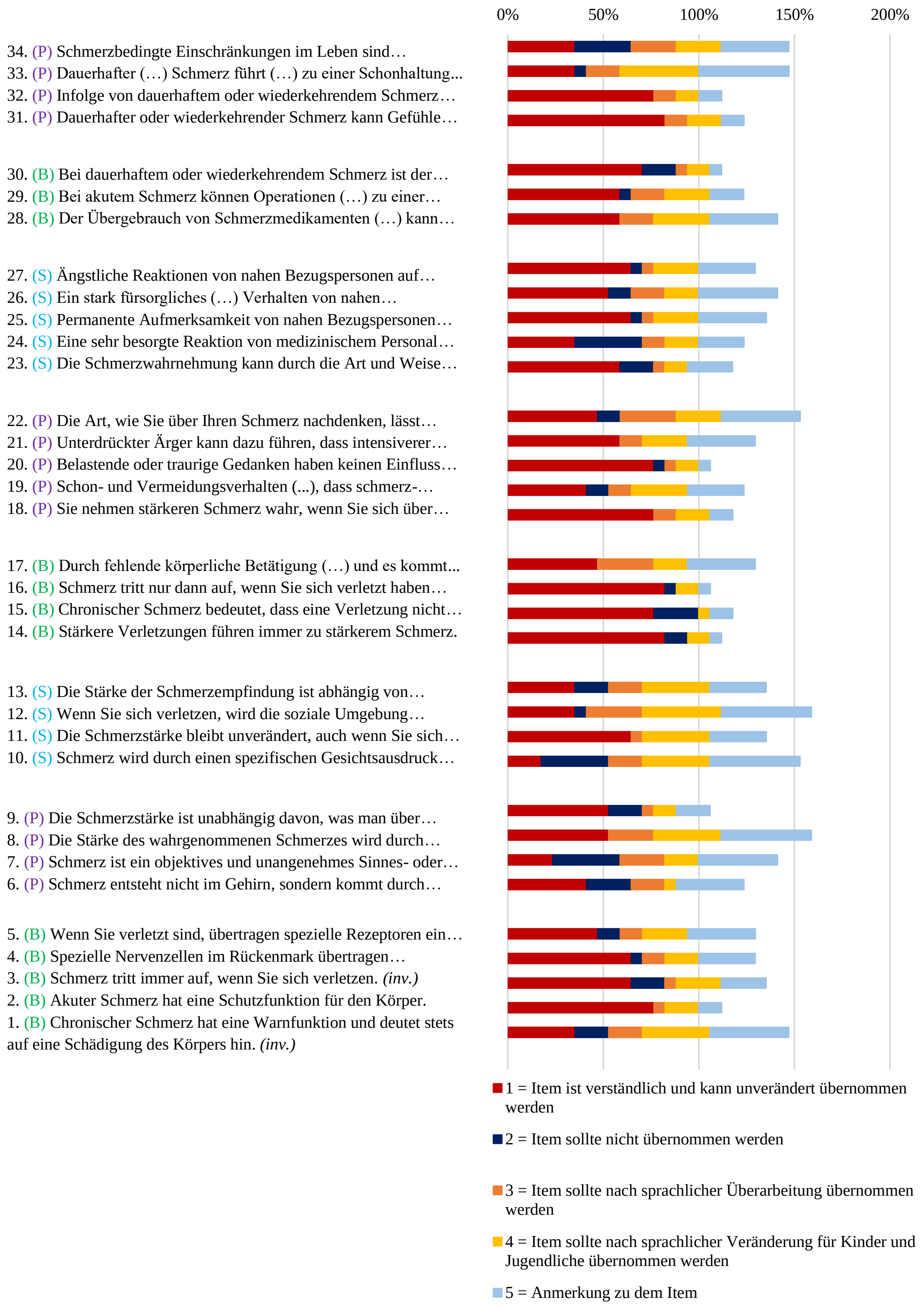

Fortsetzung Abbildung S1. Verteilung der Expert*inneneinschätzungen zu den Items der Schmerzmatrix.

Die Prozentwerte beziehen sich bei der Möglichkeit von Mehrfachantworten auf die Zahl der befragten Expert*innen, wodurch Prozentsummen von über 100% entstehen können. Eine Darstellung der ungekürzten Items ist Anhang B zu entnehmen.

(B) = Biologische Schmerzkonzepte, (P) = Psychologische Schmerzkonzepte, (S) = Soziale Schmerzkonzepte

Anhang F: Zuordnung der Expert*innenkommentare

Tabelle S2

*Zuordnung der Expert*innenkommentare zu den Ober- und Unterkategorien durch drei Rater*innen*

Kategorien	Zuordnung der Expertenkommentare in %	Beispiele
**1 Sprachliche Anmerkungen**	57.7	
1a) Sprachniveau/ Formulierung	49.6	*Statt ‚stets' ‚immer' verwenden, das ist sprachlich einfacher*
1b) Satzbau	2.7	*Ich finde den Satzbau zu kompliziert. Es sollte einfacher und ohne eingeschobenen Satz formuliert werden*
1c) Inversion	5.4	*Lieber nicht invers formulieren*
**2 Inhaltliche Anmerkungen**	28.5	
2a) Allgemeine Verständlichkeit	15.9	*Kein sozial aktives Leben führen- was soll das sein? Konkret wäre besser zu beantworten*
2b) Relevanz	1.0	*Ist sehr ähnlich zu den bereits formulierten Items*
2c) Vorwissen erforderlich	1.8	*Was sind denn psychologische Schmerzbewältigungsstrategien? Viele Patient*innen haben davon noch nie gehört*
2d) Kinder/Jugendliche vs. Erwachsene	9.8	*Schmerz wahrnehmen ist manchmal etwas schwierig für Kinder*
**3 Bewertung**	3.6	
3a) Zustimmung	0.9	*Dieses Item ist wirklich gut*
3b) Ablehnung	2.7	*Kein gutes Item*

**4 Keine Auswertung auf Itemebene möglich**[a]		
4a) Nicht auswertbar[b]	5.3	
4b) Nicht relevant für das Forschungsteam[c]	3.3	*Das ist doch wohl invers gemeint, oder?*
4c) Allgemeine Hinweise zum Fragebogen[d]	1.6	*Dopplung mit anderen Fragen, zudem viel zu viele Fragen! Reduzieren sie die Fragen insgesamt auf maximal 20!*

Anmerkungen. [a] Unter diese Kategorie fallen alle Expertenkommentare, aus denen keine Erkenntnisse bezüglich der Anpassung der Fragebogenitems gewonnen werden können. [b] Unter diese Unterkategorie fallen alle Kommentare, die Verständnisfragen seitens der Experten zu dem Item beinhalten. [c] Diese Unterkategorie beinhaltet alle Expertenkommentare, die an der eigentlichen Intention der Befragung vorbeigehen, z.B. Hinweise auf dem Forschungsteam bereits aufgefallene Fehler der Items. [d] Diese Unterkategorie beinhaltet alle Expertenkommentare, welche die allgemeine Fragebogenkonstruktion betreffen.

Anhang G: Revidierte Version der BiPS Matrix

Tabelle S3

*Revidierte Version der BiPS Matrix auf Basis der hier vorgestellten Expert*innenkommentare*

Itemnr.	Inhaltsdimenion	Inhaltbereich	Item[a]
1.	Biologisch	Art der Störung	Chronische Schmerzen haben eine Warnfunktion. (*inv.*)[b]
2.	Biologisch	Art der Störung	Akute Schmerzen haben eine Schutzfunktion für den Körper
3.	Biologisch	Art der Störung	Spezielle Nerven im Rückenmark senden Gefahrenmeldungen ans Gehirn.
4.	Biologisch	Art der Störung	Wenn man seit mehreren Monaten Schmerzen hat, wird das Gehirn empfindlicher für Warnsignale.
5.	Psychologisch	Art der Störung	Das Gehirn verarbeitet viele Details, bevor es

			entscheidet, wann man Schmerzen empfindet.
6.	Psychologisch	Art der Störung	Gedanken können die Schmerzstärke beeinflussen.
7.	Psychologisch	Art der Störung	Die Schmerzstärke ist unabhängig davon, was man über Schmerzen weiß. (*inv*.)
8.	Sozial	Art der Störung	Die Schmerzstärke ändert sich, wenn man sich mit dem/der Partner*in, der Familie oder einem/einer guten Freund*in streitet.
9.	Sozial	Art der Störung	Die Schmerzstärke bleibt gleich, unabhängig davon, wo und mit wem man gerade zusammen ist. (*inv*.)
10.	Sozial	Art der Störung	Die Schmerzstärke unterscheidet sich bei Menschen aus verschiedenen Kulturen.
11.	Biologisch	Annahmen zur Ursache	Schlimmere Verletzungen bewirken immer auch stärkere Schmerzen. (*inv*.)
12.	Biologisch	Annahmen zur Ursache	Schmerz tritt nur auf, wenn man verletzt ist oder ein Verletzungsrisiko besteht. (*inv*.)
13.	Biologisch	Annahmen zur Ursache	Wenn man sich aus Angst vor Schmerzen gar nicht oder wenig bewegt, werden Muskeln abgebaut. Das führt zu stärkeren Schmerzen.
14.	Psychologisch	Annahmen zur Ursache	Man spürt Schmerzen stärker, wenn man sich

			wegen seiner Schmerzen Sorgen macht.
15.	Psychologisch	Annahmen zur Ursache	Unterdrückter Ärger oder Trauer kann dazu führen, dass Schmerzen stärker werden.
16.	Psychologisch	Annahmen zur Ursache	Die Art, wie man über seine Schmerzen denkt, verändert die Schmerzen nicht. (*inv*.)
17.	Psychologisch	Annahmen zur Ursache	Durch eine Aufklärung über Schmerzen, können die Schmerzen schwächer werden.
18.	Sozial	Annahmen zur Ursache	Ständige Aufmerksamkeit durch den/die Partner*in, Freund*innen oder die Familie auf andauernde Schmerzen kann die Schmerzen verschlimmern.
19.	Sozial	Annahmen zur Ursache	Wenn Eltern bei einer Verletzung eines Kindes ängstlich reagieren, können die Schmerzen stärker werden.
20.	Biologisch	Konsequenzen	Durch zu viele Schmerzmedikamente können die Schmerzen dauerhaft bestehen bleiben.
21.	Biologisch	Konsequenzen	Bei akuten Schmerzen können Operationen, die der Behandlung der Schmerzursache dienen, zu einer Linderung der Schmerzen führen.
22.	Psychologisch	Konsequenzen	Wenn man chronische Schmerzen hat, kann man sich hilflos oder hoffnungslos fühlen.
23.	Psychologisch	Konsequenzen	Chronische Schmerzen führen oft zu

			Beeinträchtigungen im Alltag der Betroffenen.
24.	Sozial	Konsequenzen	Man kann trotz chronischer Schmerzen etwas gemeinsam mit Freund*innen, dem/der Partner*in oder der Familie unternehmen.
25.	Sozial	Konsequenzen	Chronische Schmerzen können dazu führen, dass man sich häufiger von Freund*innen, der Familie oder dem/der Partner*in zurückzieht.
26.	Sozial	Konsequenzen	Schmerzen können dazu führen, dass man nicht mehr arbeiten gehen kann.
27.	Biologisch	Zeitlicher Krankheitsverlauf	Akute Schmerzen haben normalerweise einen klaren Auslöser. Nach der Heilung werden die Schmerzen weniger.
28.	Biologisch	Zeitlicher Krankheitsverlauf	Wenn eine Verletzung richtig ausgeheilt ist, hat man keine Schmerzen mehr. (*inv.*)
29.	Psychologisch	Zeitlicher Krankheitsverlauf	Wenn man glücklich ist, kann das die Schmerzen verringern.
30.	Psychologisch	Zeitlicher Krankheitsverlauf	Wenn man chronische Schmerzen hat, empfindet man immer Schmerzen. (*inv.*)
31.	Sozial	Zeitlicher Krankheitsverlauf	Die Schmerzen bleiben über den Tag gleich, unabhängig davon, mit wem man den Tag zusammen verbringt. (*inv.*)
32.	Biologisch	Möglichkeiten der Kontrolle/ Behandlung	Man muss immer Medikamente einnehmen, um akute

			Schmerzen zu behandeln. (*inv.*)
33.	Biologisch	Möglichkeiten der Kontrolle/ Behandlung	Um mit chronischen Schmerzen umzugehen, muss man nicht immer Medikamente einnehmen.
34.	Biologisch	Möglichkeiten der Kontrolle/ Behandlung	Sport und Bewegung helfen dabei, chronische Schmerzen zu verringern.
35.	Psychologisch	Möglichkeiten der Kontrolle/ Behandlung	Ablenkung kann die Schmerzen verringern.
36.	Psychologisch	Möglichkeiten der Kontrolle/ Behandlung	Angenehme Tätigkeiten (z.B. Musik hören) verringern die Schmerzen nicht. (*inv.*)
37.	Psychologisch	Möglichkeiten der Kontrolle/ Behandlung	Wenn man sich entspannt, ist es einfacher mit Schmerzen umzugehen.
38.	Sozial	Möglichkeiten der Kontrolle/ Behandlung	Bei chronischen Schmerzen ist es gut, sich mit Freund*innen, dem/der Partner*in oder der Familie zu treffen und seinen Hobbies weiter nachzugehen.
39.	Sozial	Möglichkeiten der Kontrolle/ Behandlung	Eltern haben keinen Einfluss auf chronische Schmerzen der Kinder. (*inv.*)
40.	Sozial	Möglichkeiten der Kontrolle/ Behandlung	Behandlungsteams (z.B. aus Psychotherapeut*innen, Ärzt*innen, Physiotherapeut*innen, Krankenpfleger*innen) können helfen, die Schmerzstärke zu verringern.

Anmerkungen. [a] *Das Antwortformat enthält folgende, fünfstufige Likertskala: „stimme überhaupt nicht zu“, „stimme nicht zu“, „weder noch“, „stimme zu“, „stimme stark zu“.* [b] *inv. = invertiertes Item.*
